# The cisplatin-induced lncRNA PANDAR dictates the chemoresistance of ovarian cancer via regulating SFRS2-mediated p53 phosphorylation

**DOI:** 10.1038/s41419-018-1148-y

**Published:** 2018-10-30

**Authors:** Hao Wang, Lei Fang, Jing Jiang, Ye Kuang, Beidi Wang, Xiumin Shang, Peilin Han, Yue Li, Meimei Liu, Zongfeng Zhang, Peiling Li

**Affiliations:** 1 Departments of Obstetrics and Gynecology, The Second Affiliated Hospital of Harbin Medical University, No. 246 Xue Fu Road, Nangang District Harbin, 150001 China; 2 Departments of Obstetrics and Gynecology, Mudanjiang Women and Children’s Hospital, Mudanjiang, China; 3 Department of Surgery, The first affiliated hospital of Zhejiang University, Hangzhou, China; 4 Departments of Intensive Care Unit, The Second Affiliated Hospital of Harbin Medical University, Harbin, China

## Abstract

As a component of p53-dependent lncRNA (long non-coding RNA), *PANDAR* (the promoter of CDKN1A antisense DNA damage activated RNA) participates in the epigenetic regulation in human cancer. However, the involvement of *PANDAR* in cancer chemoresistance is unknown. In this study, we report that *PANDAR* serves as a negative regulator of cisplatin sensitivity in human ovarian cancer via *PANDAR*-SRFS2-p53 feedback regulation in nuclear. Our data showed that among the drugs commonly used in ovarian cancer therapy, cisplatin induces higher levels of *PANDAR* compared with doxorubicin and paclitaxel. We also proved that *PANDAR* exhibited higher expression in cisplatin-resistant ovarian cancer tissues and cells, compared with cisplatin-sensitive ones, and this expression pattern depends on wild-type p53 (wt-p53), not mutant-p53 (mt-p53). In vitro and in vivo, *PANDAR* overexpression improved cell survival rate and tumor growth in response to cisplatin, while depletion of *PANDAR* leads to a reduced tumor growth. Further investigation revealed that *PANDAR*-reduced cisplatin sensitivity was likely or partly due to the *PANDAR*-binding protein SFRS2 (arginine/serine-rich 2), a splicing factor with the ability to negative regulate p53 and its phosphorylation at Serine 15 (Ser15). This feedback regulation of *PANDAR*–SFRS2–p53 leads to a reduced transactivation of p53-related pro-apoptotic genes, such as PUMA (p53-upregulated modulator of apoptosis). In addition, in platinum-treated patients with relapsed ovarian cancer, resistant period was positively correlated with the expression of *PANDAR* and SFRS2, and inversely associated with expression of p53-Ser15 and PUMA in these clinical tissues. Last but not least, the role of *PANDAR* in chemoresistance was confirmed in patients with ovarian cancer. These findings reveal a novel regulatory maneuver of cancer cells in response to chemostress, and might shed light on overcoming cisplatin resistance in ovarian cancer.

## Introduction

Ovarian cancer (OC) continues to kill more than 150,000 women every year worldwide^1^. It is usually advanced when diagnosed. Staging is surgical. Treatment requires cytoreduction and chemotherapy. Chemotherapy is essential for the management of cancer progression^1^. However, drug resistance can lead to treatment failure^2^. Hence, a better understanding of chemoresistance in ovarian cancer therapeutics is urgently needed.

Cisplatin, the basic anticancer drug of chemotherapy, often develop drug resistance in ovarian cancer treatment^2^. To date, the mechanism of cisplatin resistance has been elusive^3^. Although the tumor suppressor p53 phosphorylation at Serine 15 (Ser15) and Serine 20 (Ser20) were identified as the key to cisplatin resistance in OC^3,4^, it still lacks a clear regulatory mechanism during this process. Serine-rich and arginine-rich proteins (SR proteins), a family of RNA-binding proteins, were initially discovered as regulators of alternative splicing^5^. Recent studies have revealed that SR proteins are involved in p53 and its phosphorylation and acetylation^6,7^. For instance, in response to ribosomal disturbances, SFRS1 (arginine/serine-rich 1) interacts with MDM2 (murine double minute 2) to inhibit p53 degradation^6^. p53 post-translational turnover is regulated by another member of SR family, SFRS2 (arginine/serine-rich 2), also called SC35 or SRFS2. SFRS2 depletion from mouse embryonic fibroblasts could result in p53 hyperphosphorylation^6^. However, whether SFRS2 regulates p53 phosphorylation in human OC remains unclear.

Long non-coding RNAs (lncRNAs), with 200–100,000 nt in size, has been found to regulate various cellular mechanisms, including cisplatin resistance^8^, through interacting with proteins and co-factors^9^. *PANDAR*, the Promoter of CDKN1A Antisense DNA damage Activated RNA, was first reported as the most upregulated p53-dependent lncRNA responding to drug-induced cell apoptosis^10^. The roles of *PANDAR* are diverse according to the cellular location and interaction partners. For instance, when bound to the SAFA (the scaffold attachment factor A) protein in cardiomyocytes, *PANDAR* regulates cellular senescence^11^. In this study, we found a matching sequence of *PANDAR* (167bp–176bp) containing 5′-CCAG-3′, which is reported as the high-affinity binding sequence recognized by SFRS2 and could now be found in all SELEX (Systematic Evolution of Ligands by Exponential Enrichment) consensus sequences and in all identified SFRS2-specific ESEs (exon-splicing enhancers)^12^. In line with these observations, we reason that whether *PANDAR* could interact with SFRS2 in OC cells.

To fill the above gaps, we studied the role of *PANDAR* in cisplatin sensitivity and discovered that cisplatin-induced *PANDAR* expression counter-regulates nuclear p53 and its phosphorylation at Ser15 via interacting with SFRS2, which in turn, attenuates cisplatin sensitivity in ovarian cancer chemotherapy.

## Results

### Inverse association between *PANDAR* expression and cisplatin sensitivity in OC

**Fig. 1:**
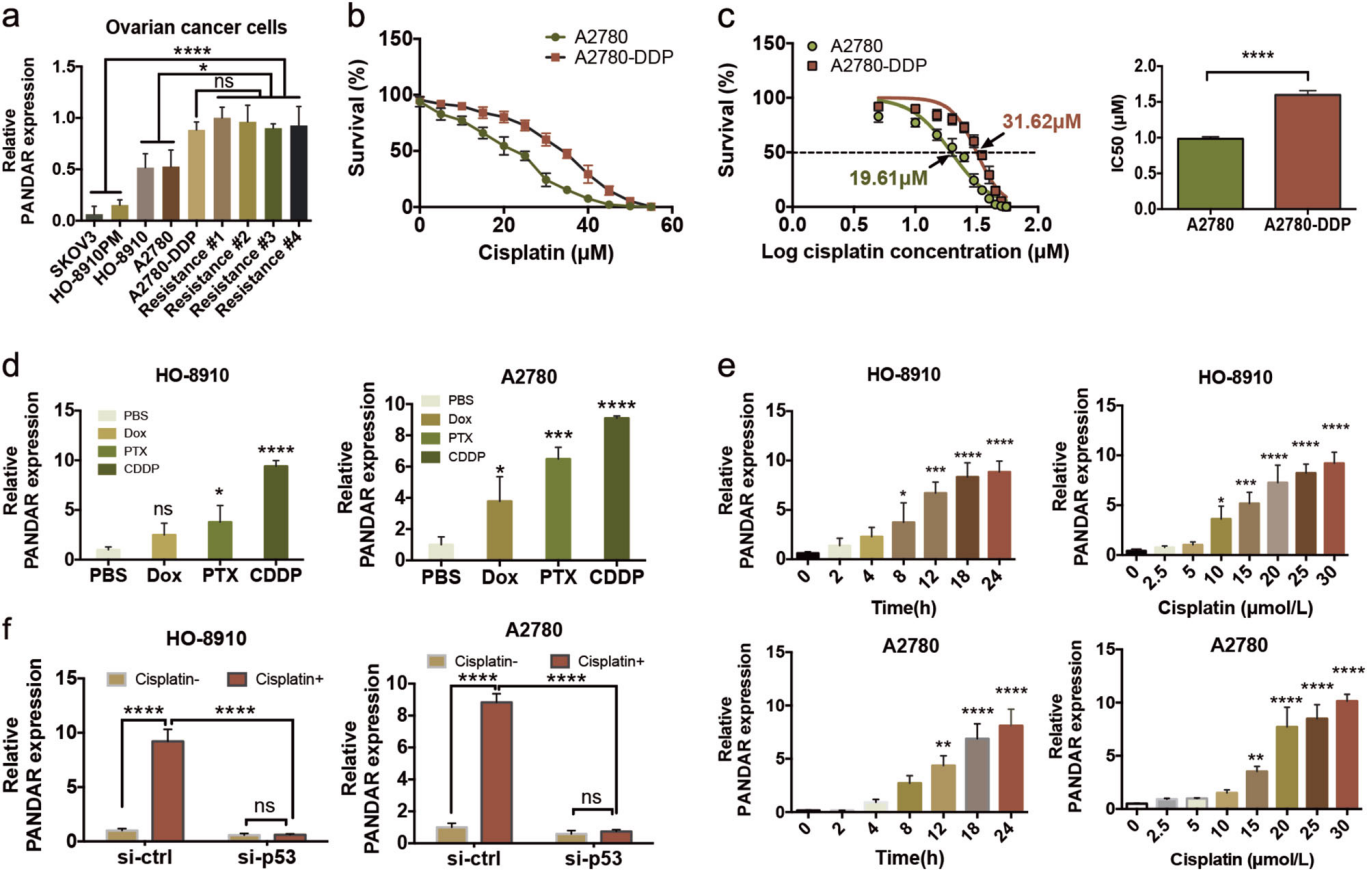
Inverse association between *PANDAR*-expression and cisplatin sensitivity in ovarian cancer cells. **a** QRT-PCR analysis of *PANDAR* expression in the chemosensitive ovarian cancer cell lines, A2780 cisplatin-resistant cell lines (A2780-DDP) and primary recurrent ovarian cancer cells of the 3rd generation. **b** CCK-8 assay of cell survival rate in A2780 and A2780-DDP cells after treated with an increasing dose (0–55 μM) of cisplatin for 24 h. **c** The half maximal inhibitory concentration (IC50) was calculated from Fig. 1b via three independent experiments using GraphPad 6.2 software. **d** QRT-PCR of PANDAR expression in A2780 and HO-8910 cells after treated with phosphate buffer saline (PBS, 5 µM), doxorubicin (DOX, 5 µM), paclitaxel (PTX, 25 nM), or cisplatin (CDDP, 20 µM) for 24 h. PBS serves as control. **e** *PANDAR* expression in A2780 and HO-8910 cells after treated with 20 µM cisplatin for indicated time (left) or treated with indicated concentrations (right) of cisplatin for 24 h. **f** *PANDAR* expression in A2780 and HO-8910 cells with *TP53* silenced after treated with or without 20 µM dose of cisplatin for 24 h. Vehicle groups were treated with PBS. PBS groups are normalized to 1. Data presents the mean ± S.D. *p < 0.05, **p < 0.01, ***p < 0.001, ****p < 0.0001, ns non-significant. Two-tailed Student’s *t*-test. *n* = 3 independent experiments

To investigate whether lncRNA *PANDAR* was associated with ovarian cancer chemosensitivity, we examined *PANDAR* expression profile in cisplatin-sensitive and cisplatin-resistant cells of OC (Fig. 1). First, we detected the expression profiles of wt-p53 and mt-p53 in OC cell lines, in which *PANDAR* expression was largely determined. Data showed that wt-p53 was positive in OC cell lines except SKOV3, and wt-p53 was only seen in the cytoplasm of A2780-DDP and HO-8910PM cells (Supplementary Fig. S1a, b), indicating that *PANDAR* roles in ovarian cancer chemoresistance could be sought among A2780, HO-8910, HO-8910PM, and A2780-DDP cell lines. We also isolated primary cells from the recurrent OC samples without p53 mutation (Supplementary Fig. S1c, Table 1), namely Resistance #1, #2, #3, #4, and then measured *PANDAR* expression level in these recurrent cells, cisplatin-resistant cell line (A2780-DDP), and cisplatin-sensitive cell lines (A2780, HO-8910, HO-8910PM, and SKOV3). Data showed *PANDAR* level was higher in resistant OC cells compare with cisplatin-sensitive cells, but there was no significance among those chemoresistant cells (Fig. 1a). Cell survival rate (Fig. 1b) and IC50 (Fig. 1c) in A2780 and A2780-DDP cell lines were measured with an increasing cisplatin treatment, validating A2780-DDP cells are more prone to survive compared with A2780 cells in response to cisplatin. These observations suggest that *PANDAR* may play a role in platinum-based resistance in OC. To confirm this, we measured *PANDAR* levels in A2780 and HO-8910 cells following treatments by chemo-drugs doxorubicin (Dox), paclitaxel (PTX), and cisplatin (CDDP), as they were commonly used in clinical ovarian cancer chemotherapeutics. We found that cisplatin induced the highest expression of *PANDAR* among other drugs (Fig. 1d) in a dosage-dependent and time-dependent manner (Fig. 1e). The induction of *PANDAR* by cisplatin was also p53 dependent (Fig. 1f). These results suggest that cisplatin-induced *PANDAR* may dictate cisplatin resistance of OC without p53 mutation.

**Table 1:** Relative expression of PANDAR, p53-Ser15, and PUMA in patients with newly diagnosed and recurrent ovarian cancer

No	Age (years)	Classification	FIGO stage	Differentiation	Platinum sensitive (N/R)	p53 mutation	PANDAR Exp. (N/R)	p53-Ser15 Exp. (N/R)	PUMA Exp. (N/R)
1	50	OCCC	IIB	N/A	Yes/ No	No	Low/ high	High/ low	High/ low
2	51	ENOC	IIIC	Poor	Yes/ No	No	Low/ high	High/ low	High/ low
3	49	ENOC	IIIA	Poor	Yes/ No	No	Low/ high	High/ low	High/ low
4	53	HGSOC	IIC	Poor	Yes/ No	No	Low/ high	High/ low	High/ low
5	80	HGSOC	IIIB	Poor	No/ No	Yes	Low/ low	Low/ low	Low/ low
6	62	HGSOC	IIIC	Moderate	No/ No	Yes	Low/ low	Low/ low	Low/ low
7	56	HGSOC	IIIC	Poor	No/ No	Yes	Low/ low	Low/ low	Low/ low

Low, below the median of the intergrated optical density. High, above the median of the intergrated optical density

*HGSOC* high-grade serous ovarian cancer, *OCCC* ovarian ovarian clear cell carcinomas, *ENOC* endometrioid ovarian cancer, *Exp* expression, *N* newly diagnosed, *R* recurrence

### *PANDAR* attenuates cisplatin sensitivity in chemosensitive OC cells

**Fig. 2:**
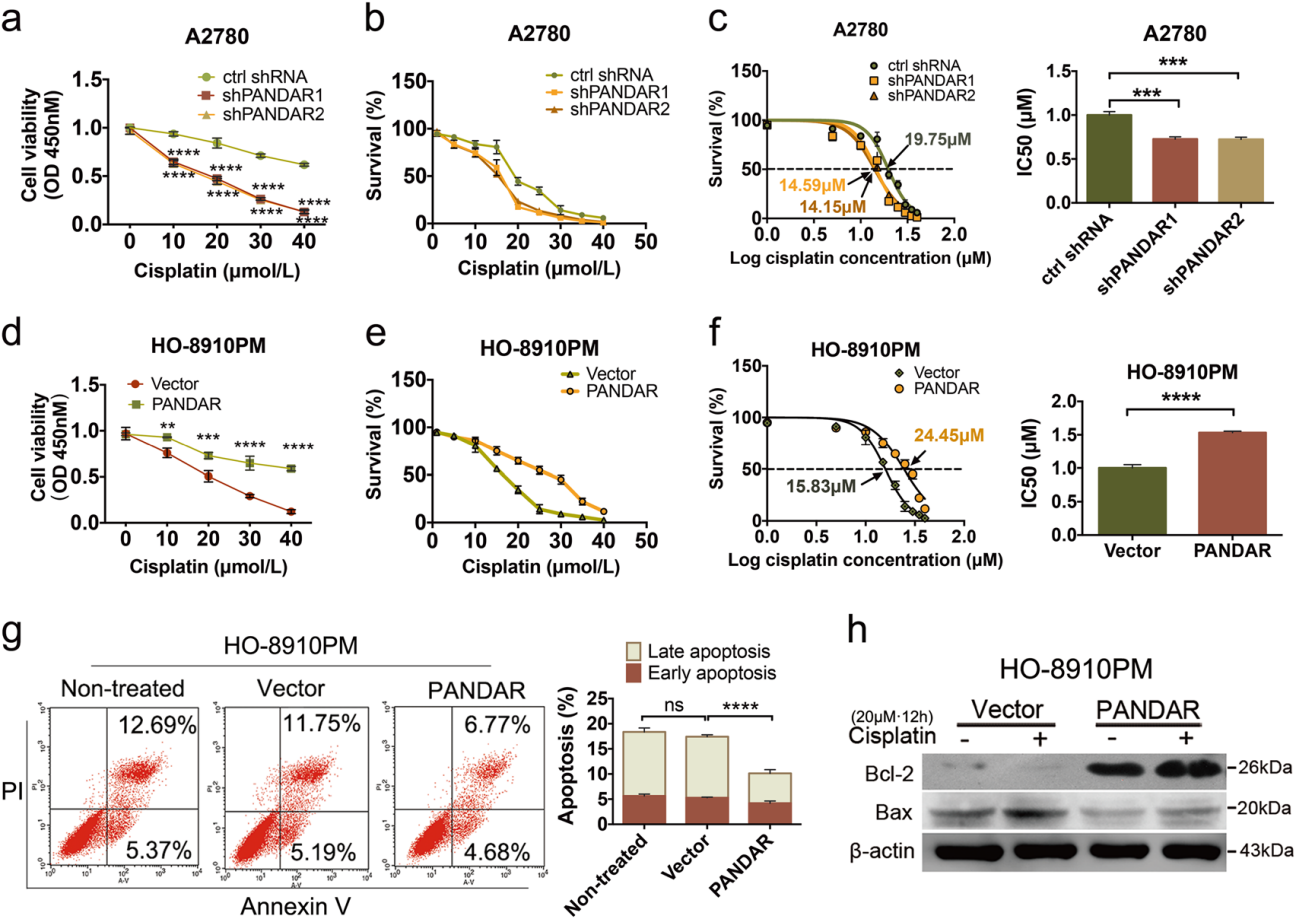
PANDAR reduces cisplatin sensitivity in chemosensitive ovarian cancer cells. **a** CCK-8 assay of cell viability in A2780-PANDAR-knockdown cells (shPANDAR) after treated with indicated doses of cisplatin for 24 h, ctrl shRNA cells serve as controls. **b** Cell survival rate was measured from **a** through three independent experiments. **c** The half maximal inhibitory concentration (IC50) was calculated from **b** using GraphPad 6.2 software. **d** Cell viability in HO-8910PM-PANDAR cells after treated with indicated doses of cisplatin for 24 h via CCK-8 analysis, HO-8910PM-Vector cells serve as controls. **e** Cell survival rate was calculated from **d** through three independent experiments. **f** The half maximal inhibitory concentration (IC50) was calculated from **e** using GraphPad 6.2 software. **g** Flow cytometry assay (FCM) of cell apoptosis in chemosensitive HO8910PM-PANDAR-overexpressing cells with or without a 24-h treatment of 20 μmol/L cisplatin. Early apoptotic population in the lower right gate is characterized with Annexin V (+) and PI (−), and late apoptotic population in the upper right gate is characterized with Annexin V (+) and PI (+). **h** Protein expression of Bax and Bcl-2 in HO-8910PM-PANDAR overexpressing cells with a 12-h treatment of 20 μM cisplatin, Non-treated Vector cells serve as controls. Vector and ctrl shRNA groups are normalized to 1. Data presents the mean ± S.D. *p < 0.05, **p < 0.01, ****p < 0.0001, ns non-significant, two-tailed Student’s *t*-test. *n* = 3 independent experiments. A-V Annexin V

To further investigate the biological functions of *PANDAR* in ovarian cancer, we created isogenic *PANDAR* Venus knock-in HO-8910PM cell line and *PANDAR* Venus knockdown (*shPANDAR*) A2780 cell line. We observed a 320-fold increase of *PANDAR* expression in *PANDAR* knock-in HO-8910PM cell line (Supplementary Fig. S2a), while a 33-fold and 3-fold decrease of *PANDAR* expression in A2780-*PANDAR*-knockdown and A2780-DDP-*PANDAR*-knockdown cell lines was detected (Supplementary Fig. S2a, b). Next, we measured cell viability, as well as survival rate and the IC50 of *PANDAR* knock-in and knock-down cells in response to cisplatin (Fig. 2a). We found that A2780-*PANDAR*-knockdown cells displayed a decreased survival ability compared with control shRNA cells (Fig. 2a, b). The IC50 was also downregulated after *PANDAR* knockdown (Fig. 2c). Conversely, HO-8910PM cells with *PANDAR* overexpression exhibited a stronger survival ability in response to high doses of cisplatin, compared with cells infected with empty vectors (Fig. 2d, e). Moreover, IC50 in *PANDAR*-overexpressing cells was almost 1.5-fold of that in Vector cells (Fig. 2f). On the other hand, apoptosis population was decreased after *PANDAR* overexpression in HO-8910PM cells (Fig. 2g), characterized with a slight downregulation of early apoptosis (Annexin V+, PI−) and a significant reduction of late apoptosis (Annexin V+, PI+), compared with non-treated cells and vector-infected cells, suggesting that *PANDAR* overexpression reduces the sensitivity of OC cells in response to cisplatin. To further reconfirm this suggestion, we detected and quantified Bax and Bcl-2 expression levels in that the increased ratio of Bax/Bcl-2 is an indicator of apoptosis initiation^13^. As expected, Bax was downregulated in *PANDAR*-overexpressed cells whereas Bcl-2 level was upregulated, especially after a 12-h cisplatin treatment (Fig. 2h). The Bax/Bcl-2 ratio upon cisplatin treatment was significantly downregulated in HO-8910PM*-PANDAR* overexpressing cells (Supplementary Fig. S2c), compared with the empty vector groups. More importantly, cisplatin-induced Bax/Bcl-2 ratio was limited in *PANDAR*-overexpressing cells, indicating that PANDAR overexpression reduces cisplatin sensitivity. These data suggest that *PANDAR* functions as an oncogene in OC cells and reduces cisplatin sensitivity.

### *PANDAR* reduces cisplatin sensitivity in a mouse model

**Fig. 3:**
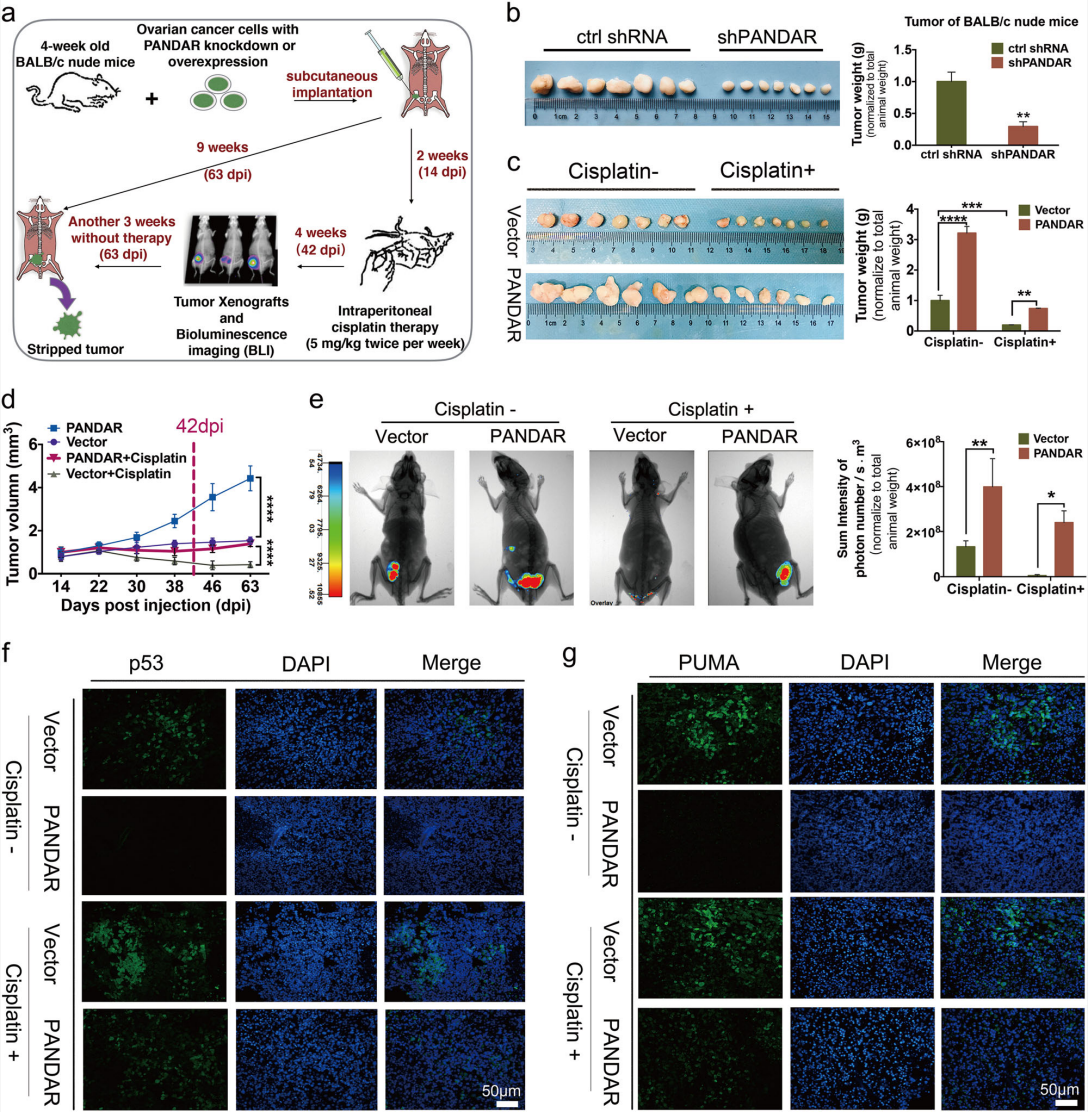
PANDAR promotes tumor growth and reduces cisplatin sensitivity in mice with tumor implantation. **a** Schematic diagram showing the process of subcutaneous tumor transplantation in mice with PANDAR over-expression or knock-down (shPANDAR) cells. **b** Subcutaneous tumor derived from mice with A2780-shPANDAR cells and -ctrl shRNA cells transplanted after 9 weeks. No treatment applied during this time. Tumor weight was normalized to total animal weight. **c** Subcutaneous tumor derived from mice with HO8910PM-PANDAR cells and -Vector cells at the 63-day post injection (63 dpi). Cisplatin treatment was applied during this time. Tumor weight is normalized to total animal weight. **d** Tumor growth curves of mice injected with PANDAR-overexpressing cells after treated with cisplatin since 14 dpi. Non-treated mice with vector cells serve as controls. **e** Bioluminescence imaging (BLI) of tumor and accordingly quantification (intensity of photon number) in BALB/c nude mice implanted with HO-8910PM-PANDAR-GFP or HO-8910PM-Vector-GFP cells at 42 dpi after cisplatin therapy. **f** Immunofluorescent staining of p53 on subcutaneous tumor sections from mice implanted with HO-8910PM-PANDAR or -Vector cells at 63 dpi after a 4-week cisplatin therapy. Nuclei are stained in blue. Scale bar: 50 µm. **g** Immunofluorescent staining of PUMA on subcutaneous tumor sections from mice implanted with HO-8910PM-PANDAR or HO-8910PM-Vector cells at 63 dpi after a 4-week cisplatin therapy. Nuclei are stained in blue. Scale bar: 50 µm. Vector group is normalized to 1. Data presents the mean ± S.D. *p < 0.05, **p < 0.01, ***p < 0.001, ****p < 0.0001, ns non-significant, two-tailed Student’s *t*-test. *n* = 7 mice per group

To substantiate the role of *PANDAR* in tumor growth and cisplatin resistance in addition to in vitro, we created mouse xenograft models via subcutaneous injections of HO-8910PM-Vector-GFP and HO-8910PM-*PANDAR*-GFP cells, A2780-ctrl shRNA and -sh*PANDAR* cells. The schematic diagram was shown in Fig. 3a. Mice implanted with *shPANDAR* cells developed smaller tumors compared with control mice (Fig. 3b). At the first 2 weeks without cisplatin treatment, mice implanted with *PANDAR*-overexpressing cells started to develop tumors as early as 5 days post injection (dpi), compared with tumor onset at 8 dpi in control mice (data not shown). After 4 consecutive weeks of cisplatin therapy starting at 14 dpi, the average volume of tumor-like nodules from mice implanted with *PANDAR*-overexpressing cells was more than twofold larger than that the control mice (Fig. 3c), regardless of cisplatin treatment. Furthermore, a strike-back growth tendency occurred after cisplatin withdrawal (63 dpi) (Fig. 3d). Besides, by utilizing bioluminescence imaging (BLI) and its analysis system at 42 dpi, alive mice with *PANDAR*-overexpressing tumor with a larger subcutaneous tumor volume was observed, compared to accordingly control mice (Fig. 3e). Concomitantly, the downregulation of p53 and its upregulated modulator PUMA were observed in *PANDAR*-overexpressing nodules by immunofluorescence staining (Fig. 3f, g) and accordingly quantification (Supplementary Fig. S2d). Overall tumor growth profile during the cisplatin therapy revealed that *PANDAR* overexpression effectively attenuates cisplatin sensitivity.

### SFRS2 is a target protein of *PANDAR* in ovarian-cancer cell nucleus

**Fig. 4:**
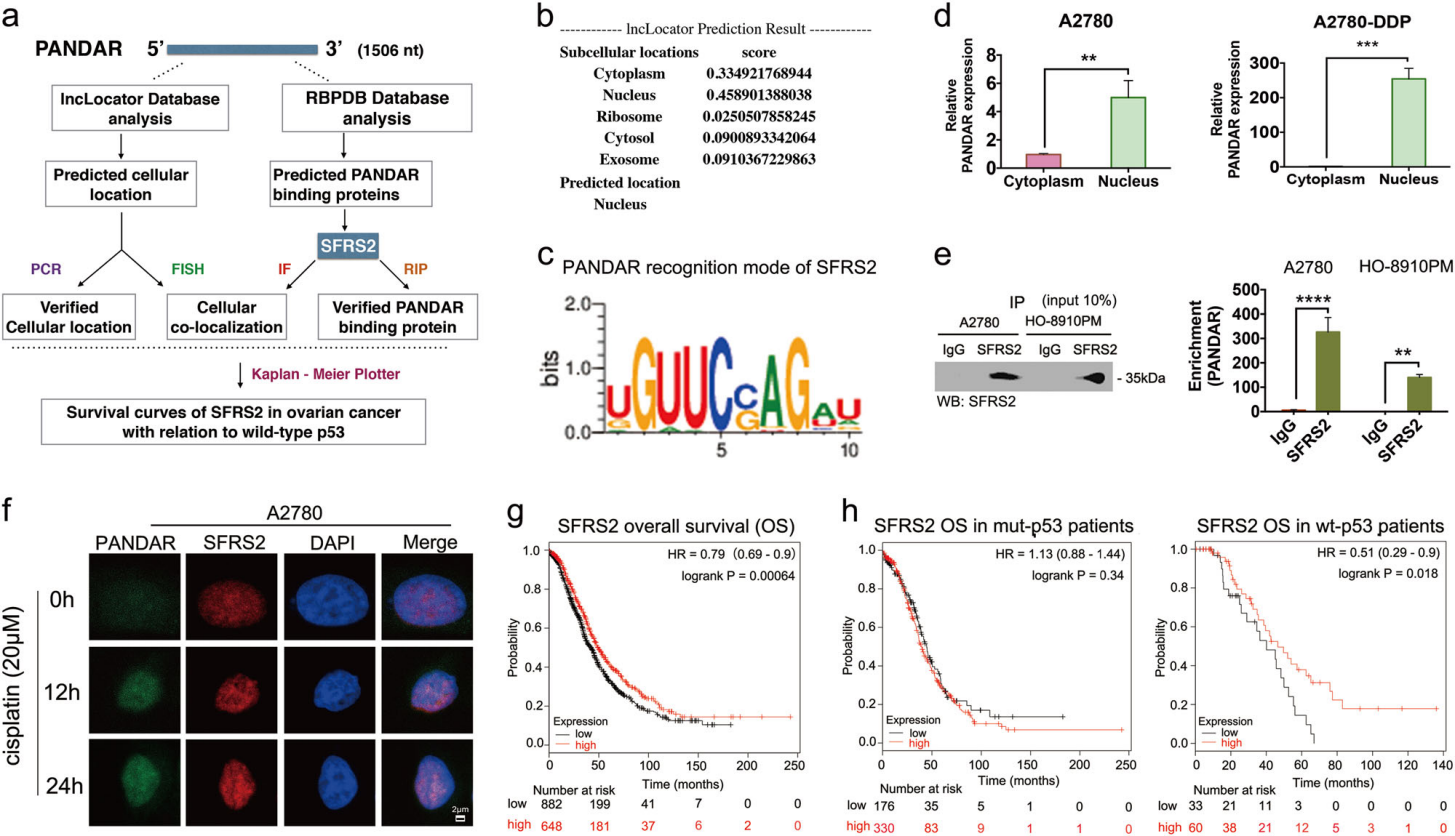
Identification of SFRS2 as a PANDAR-targeting protein in ovarian cancer cellular nucleus. **a** Schematic of prediction and consensus experiments of PANDAR-binding protein. **b** Prediction of PANDAR cellular location using lncLocator Database (http://www.csbio.sjtu.edu.cn/bioinf/lncLocator/). **c** Logo of matching sequence or motif between PANDAR and SFRS2 downloaded from RBPDB database (http://rbpdb.ccbr.utoronto.ca/experiments.php?exp_id=791). **d** QRT-PCR assay of PANDAR expression in the nucleus and cytoplasm from A2780 and A2780-DDP cells after a 24 h treatment of 20 μM cisplatin. **e** Cellular extracts from A2780 and HO-8910PM cells treated with 20 μM cisplatin for 24 h, and RNA immunoprecipitation (RIP) assay showing immunoprecipitation (IP) with control IgG or SFRS2 antibody by western blotting (left) and qRT-PCR (right) performed with isolated RNA and the following reverse transcription (RT) using primers for lncRNA PANDAR. **f** Fluorescence in situ hybridization (FISH) assay of PANDAR RNA (green) in A2780 cells distributes in discrete foci through the nucleus and cytoplasm at the beginning of treatment with 20 μM cisplatin (0 h). The foci increased and focused in the nucleus after cisplatin treatment for 12 and 24 h. Co-localization of SFRS2 protein (red) in A2780 cells via immunocytochemistry performed with SFRS2 antibodies and showed an increasing discreted foci through the nucleus after cisplatin treatment (12 h, 24 h), and is not detected in cytoplasm. Scale bar: 2 μm. **g** Kaplan–Meier Plotter of overall survival (OS) regarding SFRS2 (200753_x_at) in ovarian cancer patients (http://kmplot.com/analysis/index.php?p=service&start=1). Kaplan–Meier Plotter of SFRS2 (200753_x_at) overall survival (OS) in ovarian cancer patients with (right) or without (left) p53 mutation. Control groups are normalized to 1. Data presents the mean ± S.D. **p < 0.01, ***p < 0.001, ****p < 0.0001, two-tailed Student’s *t*-test. *n* = 3 independent experiments

To investigate how *PANDAR* attenuate cisplatin sensitivity, we sought for *PANDAR*-target proteins at the RNA-binding Proteins Database (RBPDB) and identified SFRS2 as the best candidate of PANDAR-binding proteins (Fig. 4a). First of all, SFRS2 is the top among the predicted binding proteins for PANDAR, and the nuclear location of SFRS2 accords with predicted location of PANDAR (Fig. 4b). Moreover, SFRS2 has been reported to be an inhibitor of p53 and its phosphorylation^6,7^, which might involve in PANDAR generation. The most effective evidence is that the matching sequence of 5′-CCAG-3′ within 167 bp–176 bp of *PANDAR* (Fig. 4c, Supplementary Materials), which has been reported as the high-affinity binding sequence for SFRS2 in all identified SFRS2-specific ESEs (exon-splicing enhancers)^12^. Therefore, we detected SFRS2 expression and *PANDAR* location in OC cell. As predicted in the lncLocator Database, *PANDAR* expression in nuclear is far more than that in cytoplasm either in cisplatin-sensitive (A2780) or in cisplatin-resistant (A2780-DDP) OC cell (Fig. 4d), the internal control of nuclear and cytoplasm was *U6* and *GAPDH*, respectively (Supplementary Fig. S2e). Then we detected endogenous *PANDAR* RNA co-immunoprecipitated with SFRS2 in cellular lysates from OC cells. In this part, the interaction was not detected when an isogenic IgG antibody was used, whereas a robust and specific interaction between SFRS2 and *PANDAR* was read in the isogenic SFRS2 antibody group (Fig. 4e). To explore the cellular location of this interaction, we detected *PANDAR* and SFRS2 expression in A2780 cells treated with cisplatin. Data showed an increasing co-localization of *PANDAR* and SFRS2 in nucleus over time (Fig. 4f, Supplementary Fig. S2f). To further investigate the role of this candidate protein (SFRS2) in OC, we drew Kaplan–Meier curves of SFRS2 focusing on overall survival (OS) probability. Data showed that high expression of SFRS2 might develop a better progression compared with low-expression group (Fig. 4g). Interestingly, the SFRS2-OS in OC depends on wild-type p53, but not mutant-p53 (Fig. 4h). These results indicate that SFRS2 is a PANDAR-target gene in OC, and the following function in cisplatin sensitivity might be associated with SFRS2-regulated p53.

### SFRS2-mediated p53 and its phosphorylation at Serine 15 is required for *PANDAR*-regulated cisplatin sensitivity

**Fig. 5:**
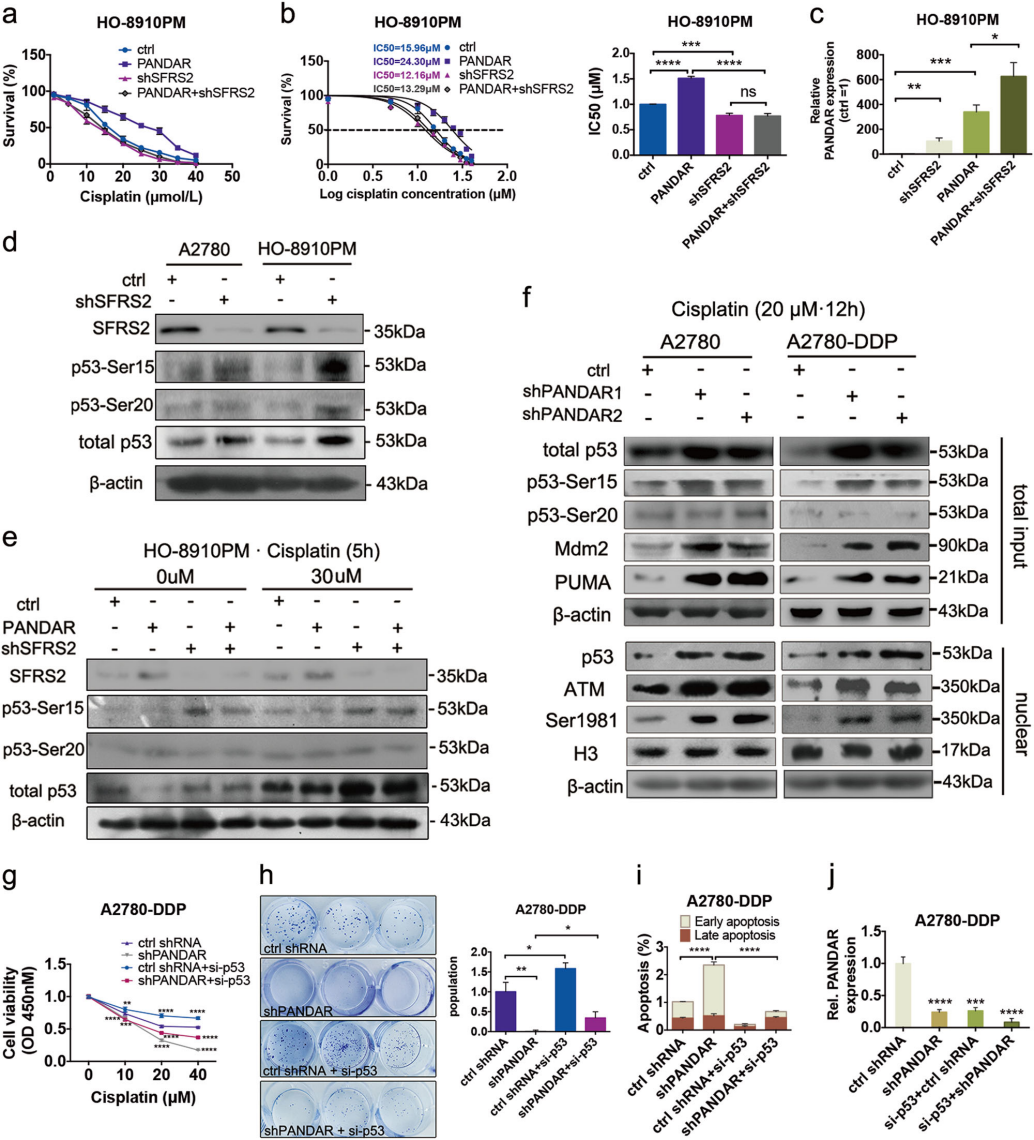
SFRS2-mediated p53 and its phosphorylation at Serine 15 is required for *PANDAR*-regulated cisplatin sensitivity. **a** CCK-8 assay of cell viability in HO-8910PM-PANDAR cells after treated with indicated doses (1–40 μM) of cisplatin for 24 h with or without SFRS2 knockdown (shSFRS2). Cell survival rate was calculated through three independent experiments. **b** Cell survival rate and the subsequent half maximal inhibitory concentration (IC50) were calculated from **a** using GraphPad 6.2 software. **c** QRT-PCR assay of PANDAR expression in HO-8910PM-PANDAR cells with or without SFRS2 knockdown (shSFRS2). **d** Protein expression performed with western blotting assay showing an increased level in p53, phosphor-p53 at Ser15 and -Ser20 in A2780 and HO-8910PM cells with SFRS2 knockdown (shSFRS2). **e** Protein expression levels of SFRS2, p53, phosphor-p53 at Ser15 and Ser20 in HO-8910PM-PANDAR cells with or without SFRS2 knockdown (shSFRS2) after 0 or 30 μM cisplatin treatment for 5 h. Non-treated cells serve as controls. **f** Proteins expression in total and nuclear fractions from A2780 and A2780-DDP cells with PANDAR knockdown (shPANDAR1, shPANDAR2) after treated with cisplatin for 12 h. H3 protein serves as nuclear control. **g** Cell viability via CCK-8 assay in A2780-DDP-PANDAR knockdown (shPANDAR) cells with or without p53 silenced after treated with indicated doses (0–40 μM) of cisplatin for 24 h. **h** Colony formation and accordingly quantification in A2780-DDP-PANDAR knockdown (shPANDAR) cells with or without p53 silenced after incubated with cisplatin for 10 days. **i** Apoptosis measurement in A2780-DDP-PANDAR knockdown (shPANDAR) cells with or without p53 silenced after incubated with 20 μM cisplatin for 24 h. **j** QRT-PCR assay of PANDAR expression in A2780-DDP-PANDAR knockdown (shPANDAR) cells with or without p53 silenced. Control groups are normalized to 1. Data presents the mean ± S.D. *p < 0.05, **p < 0.01, ***p < 0.001, ****p < 0.0001, two-tailed Student’s *t*-test. *n* = 3 independent experiments

To further explore the role of SFRS2 in *PANDAR*-regulated cisplatin sensitivity in OC, we knocked down *SFRS2* gene (*shSFRS2*) in *PANDAR*-overexpressing cells, and then detected cell survival rate and the IC50. Data showed that depletion of *SFRS2* could downregulate cell survival rate (Fig. 5a) and accordingly IC50 (Fig. 5b), regardless of *PANDAR* overexpression. This indicating that *PANDAR*-downregulated cisplatin sensitivity may be SFRS2-dependent. Interestingly, *PANDAR* expression was upregulated after SFRS2 knockdown (Fig. 5c), suggesting that SFRS2 reduced *PANDAR* expression in direct or indirect way. Recent studies have reported that SFRS2 downregulated p53 and its phosphorylation in mouse fibroblasts^6,7^. We wonder if this could be detected in OC. Next, we measured p53 and its phosphorylation at Serine 15 and Serine 20 in *SFRS2*-knockdown cells, the two important N-terminal Serine in cisplatin resistance of OC^4^. Indeed, after *SFRS2* knocked down, p53 expression and p53 phosphorylation at Serine 15 (p53-Ser15) were significantly induced compared with control cells, whereas p53-Ser20 was slightly increased (Fig. 5d). We next focused on the p53 and p53-Ser15 and -Ser20 in *PANDAR*-overexpressing cells with or without *SFRS2* knockdown. Data showed that the levels of SFRS2 and p53 reduced in *PANDAR*-overexpressing cells compared with control group (Fig. 5e), although the alteration of p53-Ser15 level was not in a very significant way, the downregulated level of p53 and p53-Ser15 by *PANDAR* was rescued when *SFRS2* knocked down (Fig. 5e). Whereas p53-Ser20 level changed not so much among all these groups (Fig. 5e). These results above indicate that *PANDAR*, as a p53-dependent lncRNA, may counter-regulate SFRS2-related p53 expression and p53-Ser15 to mediate cisplatin sensitivity. To confirm this suggestion, we detected p53 and p53-related pro-apoptotic genes expression in *PANDAR*-knockdown cells. Indeed, p53 and p53-Ser15 was significantly upregulated after *PANDAR* knockdown, either in cisplatin-sensitive cells (A2780) or cisplatin-resistant cells (A2780-DDP) (Fig. 5f, accordingly quantification in Supplementary Fig. S2g). Meanwhile, the p53-mediated apoptotic genes were upregulated, such as PUMA and Mdm2 (murine double minute 2) (Fig. 5f), *BAX* (BCL2 associated X) and *NOXA* (NADPH oxidase), but this induction drew back after p53 was silenced (Supplementary Fig. S3a). Data showed a further activation of these p53-activated genes after PANDAR knock-down. Given that these apoptosis genes transcription was partly due to p53-Ser15 in nuclear^14^, we detected nuclear p53 and the specific phosphatase ATM (ataxia-telangiectasia mutated protein kinase), in which the monomerization and its autophosphorylation at Serine 1981 are critical steps to p53 phosphorylation at Ser15^15^. As is shown in Fig. 5f, nuclear p53, ATM and its autophosphorylation at Ser1981 were all upregulated in *PANDAR*-knockdown cells, compared with control cells. These data suggest that *PANDAR* reduced cisplatin sensitivity may be partly due to SFRS2-downregulated p53 and its associated apoptotic genes transcription. To verify this hypothesis, we silenced p53 in *PANDAR* knockdown cells and then measured cell viability with an increasing cisplatin treatment. As shown in Fig. 5g, cisplatin sensitivity in cisplatin-resistant cells (A2780-DDP) was upregulated when *PANDAR* knocked down, but this was rescued after p53 was silenced. Similarly, colonies of A2780-DDP cells after a 10-day cisplatin treatment were dramatically reduced after *PANDAR* knockdown, while this ability of resistance to cisplatin regained when p53 silenced simultaneously (Fig. 5h). Apoptosis (Supplementary Fig. S3b) and accordingly analysis (Fig. 5i) also exhibited a recovered ability of resistance to cisplatin in A2780-DPP cells after p53 silenced in PANDAR-knockdown cells. Besides, *PANDAR* level was also downregulated in p53-silenced cells (Fig. 5j). p53 expression was upregulated in *PANDAR*-overexpressing cells and was downregulated in *PANDAR*-knockdown OC cells (Supplementary Fig. S3c). The above data indicate there may be a feedback loop between *PANDAR* and p53, that *PANDAR* counter-regulated the level of SFRS2-associated p53 and p53-Ser15, leading to a reduced transactivation of apoptosis genes expression in OC.

### The feedback loop between *PANDAR* and p53 contributes to the clinicopathology of cisplatin resistance in OC patients

**Fig. 6:**
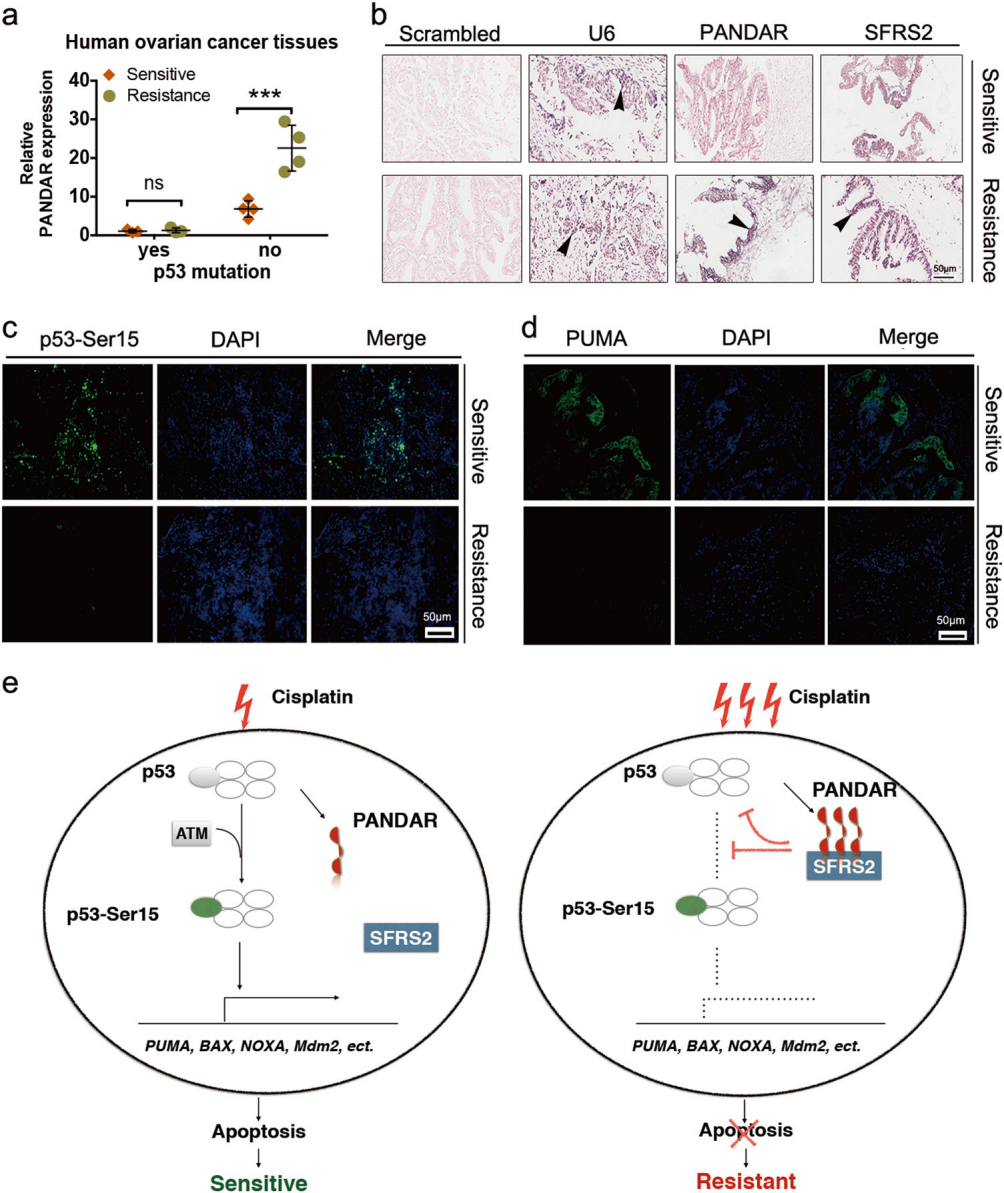
The feedback profile of *PANDAR–*SFRS2–p53 in ovarian cancer patients with chemoresistance and recurrence. **a** QRT-PCR quantification of PANDAR expression in tissues from ovarian cancer patients collected at newly diagnosed stage (Sensitive) and after disease progression during platinum-based therapy (Resistance). P53 missense mutation is in accord with post-surgical pathology reports. Data presents the mean ± S.D. *n* = 7 patients determined by Student’s *t-*test. ***p < 0.001, ns, non-significant, p53 mutation, positive mutant-p53 more than 80%. **b** LNA ISH analysis of lncRNA PANDAR with LNA probes and IHC assay of SFRS2 protein with SFRS2 antibody in matched ovarian cancer tissues before platinum-based therapy (Sensitive) and after disease progression during platinum-based treatment (Resistance). Representative LNA ISH and IHC images are shown. Scrambled groups serve as negative controls. U6 groups serve as positive controls. Black arrow head indicates the positive expression of RNA or protein. Scale bar: 50 μm. **c**, **d** Representative images of p53-Ser15 (c) and PUMA (d) immunofluorescent staining on tissues from patients at platinum-sensitive stage and after platinum-resistant ovarian cancer diagnosis. Scale bar: 50 µm. Data presents the mean ± S.D. *n* = 10 independent fields per group determined by Student’s *t*-test. **p < 0.01, ***p < 0.001. **e** Schematic model of underlying mechanisms regarding cisplatin resistance. With the sequential stimulation of cisplatin, a certain amount of p53-dependent *PANDAR* is upregulated to interact with SFRS2, leading to a reduction of p53 and its phosphorylation at Ser15, which in turn inactivated p53-mediated genes expression, such as *PUMA, BAX, NOXA, Mdm2*, and downregulated subsequent apoptosis

We further examined *PANDAR*-mediated chemoresistance in patients by tissue biopsy from 25 OC patients under platinum-based neoadjuvant and adjuvant therapy followed by complete surgical resection. Unfortunately, seven patients relapsed after the cisplatin-based or its analog carboplatin-based chemotheraputics within 6 months (Table 1), in which chemoresistance was identified as the leading cause^16^. The *PANDAR* expression regarding p53 mutation was measured in specimens from these seven patients before and after recurrence (Supplementary Fig. S4a). Data showed that *PANDAR* expression in OC tissues depends on wt-p53, but not mutant-p53, regardless of platinum-sensitivity. As is quantified by qRT-PCR, 4 of these 7 recurrent patients without p53 mutation exhibited higher levels of *PANDAR* in platinum-resistant cancer tissues compared with their newly diagnosed periods (platinum-sensitive) (Fig. 6a). Next, we examined the interplay among *PANDAR*, SFRS2, p53-Ser15, and PUMA in OC tissues collected from their sensitive and resistant periods, separately. Data showed that four patients with wt-p53 exhibited higher levels of *PANDAR* and SFRS2 (Fig. 6b) and lower levels of p53-Ser15 (Fig. 6c) and PUMA (Fig. 6d) in their resistant tissues, compared with their sensitive tissues from newly diagnosed periods. TEM images on cancer tissue biopsies also showed a better condition of cancer tissue in platinum-resistant period, compared with platinum-sensitive tissues (Supplementary Fig. S4b). These results are translational and indicated the significance of *PANDAR* and SFRS2 co-regulated p53-mediated apoptosis in clinical chemoresistance. Accordingly, we propose a schematic model of underlying mechanism regarding cisplatin resistance (Fig. 6e).

## Discussion

In this study, we identified cisplatin upregulates p53-dependent lncRNA *PANDAR* in OC cells, which is responsible for the transition from chemorsensitivity to chemoresistance upon cisplatin treatment. The mechanism involved in this transition is based on a regulatory feedback loop of *PANDAR*–SFRS2–p53 in nuclear. This feedback regulation may cast a promising target for future advances in ovarian cancer chemotherapy.

In this feedback regulation, we demonstrated that SFRS2 deregulates p53 and its phosphorylation. Given that ATM (ataxia-telangiectasia mutated protein kinase), ATR (ATM-related and Rad3-related protein kinase), and DNA-PKs (DNA-dependent protein kinase catalytic subunit) are the specific phosphatases in DNA damage-induced p53 phosphorylation at Serine 15^15^, it becomes a compelling question for the further to investigate whether SFRS2 has a role in the activation of those phosphatases.

Based on the wild-type *TP53* status, lncRNA *PANDAR* acts as an oncogene to promote cisplatin resistance in OC. Hence, choosing the appropriate cell lines with wild-type p53 protein is the first step in this study. Wild-type p53 was detected in OC chemosensitive cell lines (A2780, HO-8910, HO-8910PM), and the chemresistant cell line (A2780-DDP) in cellular nucleus, partly in cytoplasm, except SKOV3, which was p53 null as reported^17^ and showed in this study (Supplementary Fig. S1a, b). While two of those OC cell lines, namely HO-8910PM and A2780-DDP, also expressed with mutant-p53 in cytoplasm, indicating that p53 mutation occurs in these two cell lines. However, *PANDAR* functions involved in the feedback regulation of p53 in HO-8910PM and A2780-DDP were in accord with the other cell lines without p53 mutation (Figs. 2 and 5). This accordance may due to wild-type p53 expression in nucleus of these cell lines. It needs further investigation that if the co-expression of mutant-type and wild-type p53 in ovarian cancer cells was due to the alternative splicing by the splicing factor SFRS2.

The involvement of p53 in DNA damage resistance in OC is largely depended on *TP53* status and tumor histologic type^18–20^. In high-grade serous ovarian cancer (HGSOC), for example, *TP53* mutation occurs in approximately 95% of this kind of epithelial ovarian tumor and thus leads to a poor prognosis and a high rate of chemoresistance^21^. While ovarian clear cell carcinoma (OCCC) has relatively normal genomes with rare *TP53* mutations^22^, and are less sensitive to chemotherapy^23^. Therefore, a better understanding of wild-type p53 in regulating ovarian cancer may shed light on more effective chemotherapeutics. Recently, new treatments targeting DNA damage repair has recently opened the door in OC^16^, especially for modulators of p53-related mechanisms^24,25^. The previous studies have identified p53 phosphorylation at Ser15 and/or Ser20 increased the ability of p53 to facilitate cisplatin-induced apoptosis in OC^4^. Until now, only two proteins have been reported in human OC resistance to cisplatin via regulating p53 and its post-transcriptional modification, namely AKT2^4^ and in this paper, SFRS2. The cellular location and the consensus mode for these two proteins has common features in regulating cisplatin resistance. In OC cells, wild-type p53 and its phosphorylation at Ser15 were both downregulated by AKT2^4^ and SFRS2, but only SFRS2 involves in p53 feedback regulation (Fig. 6e). Moreover, the dependent on wild-type p53 status of SFRS2 role in OC progression (Fig. 4h) was first determined in this study. In 1999, Elizabeth and colleagues suggested that SFRS2 might function as a tumor suppressor gene in OC^26^. This suggestion was confirmed in this study that the high expression level of SFRS2 developed a better overall survival (OS) probability in patients with OC, compared with its low expression level group (Fig. 4g). However, in the DNA damage response to cisplatin, SFRS2 was identified as an oncogene interacting with lncRNA *PANDAR* to promote cisplatin resistance in ovarian cancer cells (Fig. 6e). This discrepancy may due to the feedback regulation of *PANDAR* and p53 induced by cisplatin.

In the past years, the interaction of SFRS2 with lncRNAs in cancer cellular nucleus has been reported^27^, but it still lacks a statement that how this protein could identify lncRNAs in human cancer cell. Our study filled this gap and revealed the matching sequence of 5′-CCAG-3′ in the lncRNA PANDAR (Fig. 4c, Supplementary Materials) on the basis of the high-affinity binding consensus sequence 5′-SSNG-3′ (S = C/G) for SFRS2 discovered by Daubner et al.^12^.

The function profile of lncRNA *PANDAR* can vary within different cell types and diseases. In breast cancer, for instance, *PANDAR* has been shown to negatively regulate cell apoptosis^28^, while in non-small cell lung cancer (NSCLC), *PANDAR* positively regulates cell apoptosis^29^. In this study, *PANDAR* attenuates cisplatin-induced apoptosis and IC50 in OC cells (Fig. 2), this pro-survival role of PANDAR in OC is also shown in other types of epithelial cancers, such as in clear cell renal cell carcinoma and bladder cancer, where *PANDAR* upregulation is associated with poor prognosis and tumorigenesis^30–34^. Inspired by these observations, our study also provided a rationale that tumor heterogeneity serves as an inherent property of lncRNA^35^. Besides, PANDAR location in ovarian cancer cell also varies with the extended response time during cisplatin treatment (Fig. 4f, Supplementary Fig. S2f), further investigation might focus on how this dynamic cellular location move forward.

To our knowledge, this is the first time a generic lncRNA feedback profile has been investigated in order to identify the features of wild-type p53 post-translational modifications in drug-induced chemoresistance in cancer therapy. This study also highlights the importance of seeking for the high-affinity matching sequence of lncRNA for RNA-binding proteins in molecular mechanism exploration. Last but not least, the insights of this study provide a rationale to develop *PANDAR* as a chemoresistant biomarker and a target for molecular therapy in OC despite the mutation of p53.

## Materials and Methods

### Reagents

Cisplatin used for this study was provided by the 2nd Affiliated Hospital of Harbin Medical University.

### Patients

All biospecimens used in this study were provided by the 2nd Affiliated Hospital of Harbin Medical University. All specimens were obtained with informed consent under the approval of the Harbin Medical University of Medicine Institutional Ethics Review Board protocol.

### Mouse colonies

Four-week-old female BALB/c nude mice used in this study were purchased from SLAC Laboratory Animal CO. LTD (Shanghai, China). Mice were housed under pathogen-free at the animal facility of the 2nd Affiliated Hospital of Harbin Medical University. All study protocols were approved by the Institutional Animal Care and Use Committee at the 2nd Affiliated Hospital of Harbin Medical University.

### Tumor xenografts and bioluminescence imaging

The previously described HO-8910PM-Vector and HO-8910PM-PANDAR cells expressing green fluorescent protein (GFP)^36,37^ were injected subcutaneously into the lower abdominal flank of BALB/c nude mice. Two weeks after the HO-8910PM-PANDAR cell injection, mice were treated with PBS (cisplatin-) or cisplatin (5 mg/kg) by intraperitoneal injection twice a week^38^. Four weeks after cisplatin treatments, animals were housed with no disturbance for another 3 weeks. The subcutaneous tumor size was measured by a caliper every 4 day. At the 42-day post injection (42 dpi), subcutaneous tumor in alive mice were observed using a BLI system. Briefly, in vivo imaging based on GFP fluorescence detection (excitation: 488 nm, emission: 510 nm) was captured by a Kodak Image Station 4000 Multi-Modal Imaging System (IS4000MM) equipped with an X-ray unit or a Kodak Image Station 2000 (TAMAR-Laboratory Supplies Ltd. ISRAEL)^39^. After killing, subcutaneous tumors were dissected out at 63 dpi. The stripped tumor weight was measured using an electronic balance and the volume was determined by caliper measurements of tumor dimensions using the prolate ellipsoid geometric model: (length × width^2^)/2^29,40^.

### Cell culture studies

Human ovarian cancer cell lines SKOV3, HO-8910, HO-8910PM, A2780, and cisplatin-resistant cell line A2780/DDP were obtained from the Chinese Academy of Sciences Committee on Type Culture Collection Cell Bank (Shanghai, China). Recurrent ovarian cancer cells were obtained from patients with postoperative pathological confirmation (Table 1 No.4–7, Supplementary Fig. S1c). All cells were cultured in RPMI (Roswell Park Memorial Institute) 1640 medium supplemented with 10% fetal bovine serum (Coring Cellgro) and 1% penicillin/streptomycin (Beyotime, Shanghai, China) in a humid incubator containing 5% CO_2_ at 37 °C.

### Lentivirus infection and stable cell line generation

Lentivirus containing overexpressed lncRNA PANDAR or shRNA that knocks down human PANDAR were constructed by subcloning the synthesized PANDAR open reading frame (ORF) into the pLVX or pLVX-GFP vector. Constructs were confirmed by sequencing. Lentivirus was produced using 293T cells, and viral packaging was conducted using ViraPower Lentiviral Packaging mix (Invitrogen, K497500). The packed virus was concentrated by an ultracentrifugation/a Lenti-X concentrator (Clontech, 631231). PANDAR overexpression or knockdown efficacy was confirmed by quantitative real-time PCR (Supplementary Fig. S2). 1 × 10^8^ TU/mL of Lentivirus, 10 μg/mL Polybrene, and 1 mL Enhanced Infection Solution (Eni.S) were infected 1 × 10^5^ cells per well on 6-well plates. To establish stable cell lines containing overexpressing or knockdown PANDAR, after lentivirus infection, cells were selected by adding 1 μg/mL puromycin for 48 h. PANDAR shRNA sequences are provided in Supplementary Table 2. Lentivirus concentrations were chosen based on preliminary studies.

### Small interfering RNA and DNA plasmids transduction

Small interfering RNA (siRNA) and p53 (p. Arg273His) DNA plasmids were transduced into ovarian cancer cells using siRNA Transfection Reagent (Cat. no. sc-29528; Santa Cruz, Biotechology, Inc.) and Lipofectamine 2000 CD Transfection Reagent (Cat. no.12566014; Invitrogen Thermo Fisher Scientific Inc.) according to manufacturer′s protocol. The Stealth RNAi^TM^ negative control duplex (cat. no. 12935–200) and siRNA duplex oligoribonucleotides targeting human p53 (Cat. no. 13750047) were obtained from Ambion (Thermo Fisher Scientific, Inc.).

### Subcellular fractionation location

Separation of the nuclear and cytosolic RNA fractions was performed using the Cytoplasmic & Nuclear RNA Purification Kit (Norgen Biotek Corp.; Thorold, Canada) according to the manufacturer’s instructions.

### Quantitative real-time PCR

Total RNA from fresh clinical samples or ovarian cancer cells was dissolved in TRIzol (Cat. No. 15596018, Invitrogen Thermo Fisher Scientific Inc.). Separated nucleoplasmic RNA was extracted from cancer cells by a Cytoplasmic & Nuclear RNA Purification Kit (Cat. No. 21000, Norgen Biotek Corp, Thorold, Canada). RNA was synthesized to cDNA using a Transcriptor First Strand cDNA Synthesis Kit (Cat. No. 04 879 030 001, Roche Molecular Systems, Inc.). Real-time PCR was performed using SYBR green reagants (Cat. No. 11 418 033 001, Roche Molecular Systems, Inc.) in a real-time PCR system (Bio-Rad). GAPDH and U6 were used as internal/ cytoplasmic and nuclear RNA control, respectively. Data were analyzed using a LightCycler 96 Instrument software (Roche Diagnostics Corporation, Indianapolis, IN, USA). Relative gene expression was calculated using the 2^−ΔΔCt^ method^41^. Primer sequences are shown in Supplementary Table 2.

### Immunofluorescent staining

Cultured cells/neurons were fixed in 4% paraformaldehyde, permeabilized with 0.2% Triton X-100, and processed for immunostaining as described previously^42^. Fluorescent microscopic images were captured on an Axiovert 200 inverted microscope (Zeiss, Thornwood, NY) equipped with a cooled CCD camera (SensiCam; Cooke, Auburn Hills, MI). Images to be directly compared were processed in an identical manner with Slidebook imaging software (Intelligent Imaging Innovations, Denver, CO) and Adobe Photoshop (version 7.0.1. or CS2 9.0.2; Adobe Systems, San Jose, CA).

### Immunohistochemistry, LNA-based in situ hybridization, fluorescence in situ hybridization, and immunocytochemistry

For immunohistochemistry (IHC), paraffin embedded sections were deparaffinized, rehydrated, followed by antigen retrieval. After primary and secondary antibody (listed in Supplementary Table 1) incubation, the slide was finally incubated with diaminobenzidine (DAB) (Dako, USA), and counterstained with hematoxylin (Sigma Chemical Co, USA).

LNA-based in situ hybridization (LNA ISH) was performed by using miRCURY LNATM miRNA ISH Optimization Kit (Exiqon, Denmark) as previously described^43^ with minor modification. Briefly, the sections were deparaffinized and deproteinated using proteinase K (15 μg/mL, Roche) at 37 °C for 10 min. The endogenous peroxidases were blocked in 1% H_2_O_2_ for 30 min, and sections were pre-hybridized at 62 °C for 30 min in formamide-free Exiqon ISH buffer (Exiqon, Denmark). Sections were then hybridized with DIG-labeled LNA probes (listed in Supplementary Table 2) for lncRNA PANDAR (50 nM) at 62 °C overnight. Slides are then stringently washed, incubated with alkaline phosphatase-conjugated anti-DIG Fab fragments (Roche, USA) for 60 min and then detected by NBT/BCIP reagent (Invitrogen, USA). Sections were finally counterstained with nuclear fast red staining solution (Sigma Chemical Co, USA).

High resolution images were captured with an Aperio ScanScope AT Turbo (Aperio, USA) equipped with Aperio ImageScope software (Aperio, USA). Assessment of the staining was based on the staining intensity and the percentage of positively stained cells using Image-Pro Plus 6.0 software (Media Cybernetics, Inc., USA).

Fluorescence in situ hybridization (FISH) and Immunocytochemistry was performed as previously described^44^. To prepare the antisense probe of lncRNA PANDAR, the cDNA fragment was amplified by using primers (5′-ctgcccagaagcaaacaggactc-3′ and 5′-tttgggagaccgaggcagacaga-3′) and was subcloned to pEASY®-T1 Cloning Vector (TransGen Biotech Co., Ltd, Beijing, China). Then A digoxigenin (DIG)-UTP labeled RNA antisense probe was synthesized using a DIG RNA labeling kit (Roche, Germany) according to the manufacturer’s instructions. The A2780 and HO-8910 cells were cultured on 24-well chamber slides and fixed with 4% PFA for 20 min at RT. was performed in hybridization buffer. Hybridization was performed at 60 °C overnight in hybridization buffer with probes added at the final concentration 400 ng/mL after prehybridization at 60 °C for 1 h. The slides were washed in PBS three times and incubated with the anti-digoxigenin fluorescein Fab fragments (Roche) diluted 1:200 for 4–5 h at RT. After washing with PBS for five times, the slides were blocked with 5% BSA in PBS for 1 h, and Anti-SC-35(SFRS2) antibody (Abcam) was diluted 1:200 in blocking buffer and incubated at 4 °C overnight. Next that, the slides were incubated with Alexa Flour568 (Invitrogen) at 37 °C for 1 h, washed with PBS and counterstained with DAPI for 10 min at RT. The signals were imaged with using a fluorescence microscope (Confocal).

### RIP assays

RIP experiments were performed using a Magna RIPTM RNA-binding Protein Immunoprecipitation Kit (Millipore) according to the manufacturer’s instructions. The SFRS2 antibody for RIP assays was obtained from Abcam Biotechnology.

### Nucleoplasmic protein separation

The Nucleoplasmic protein was obtained and separated from ovarian cancer cells using NE-PER Nuclear and Cytoplasmic Extraction Reagents (Cat. No. 78833; Thermo Fisher Scientific Inc.) according to the manufacturer’s protocol.

### Western blotting

Total protein lysates from cells were lysed in RIPA lysis buffer (Cat. No. P0013B, Beyotime, Shanghai, China) supplemented with complete Protease Inhibitor Cocktail (Cat. No. B14000, Biotool, Switzerland). Protein concentration was determined using a bicinchoninic acid (BCA) kit (Cat. No. 23227, Thermo Fisher Scientific Inc.). Samples were heated and reduced and separated on polyacrylamide gels. Separated proteins were transferred to PVDF membranes. After immunoblotting with primary and secondary antibodies with HRP conjugations, blots were reacted with enhanced chemiluminescence (ECL) reagent (Cat. No. RPN2232, GE Healthcare, Jiangsu, China). Protein semiquantification was determined using densitometry by Image J. Information of antibodies is shown in Supplementary Table 1.

### Apoptosis assay

Apoptosis was examined using FITC/ PI Annexin V Apoptosis Detection Kit I (Cat. No. 556547, BD Pharmingen) or PE/ 7-AAD Annexin V Apoptosis Detection Kit I (Cat. No. 559763, BD Pharmingen) according to the manufacturer’s protocol.

### CCK-8 assay

Cell viability was measured using the Cell Counting Kit (CCK-8/WST-8) (Cat. No. CK04, DOJINDO, Japan). Briefly, cells were plated at a density of 5 × 10^3^ cells per well on 96-well plates and subjected to indicated different treatments. Following a 24-h incubation at 37 °C, cells were incubated for an additional 3 h with CCK-8 reagent. Cell viability was read at 450 nm on a multi-label plate reader (Bio-Rad) based on color changes due to the formation of formazan product.

### Clonogenic assay

After indicated treatments, 100 number of tumor cells were plated in 12-well-plates to generate single colonies. After incubated 10 d at 37 °C, cells were fixed in 4% paraformaldehyde for 30 min, followed by 1% crystal violet staining for 15 min. After washed by water for three times, samples were photographed, and the number of visible colonies was counted by ImageJ software.

### Transmission electron microscopy

Cells were fixed with 2% paraformaldehyde and 2% glutaraldehyde in 0.1 M phosphate buffer (pH 7.4) and then post-fixed with 1% OsO_4_ for 2 h. Cells were dehydrated using a gradient series of ethanol (30, 50, 70, 90, and 100%). Cell were then incubated with LR White resin (Sigma, 62661) twice for 1 h, and subsequently embedded in LR White resin^45^. The solidified blocks were cut into 60-nm sections and stained with uranyl acetate and lead citrate. Samples were observed and imaged under a transmission electron microscope (TEM, Hitachi H-7600; Hitachi High-Technologies Corporation, Tokyo, Japan). Ten fields were selected by the presence of cytoplasm shrinkage, nuclear membrane shrinkage and/ or nuclear chromatin in the outer nuclear layer gathered towards the center with uneven distribution, and the results were averaged.

### Statistical analyses

All data were exported to GraphPad Prism v6.2 (GraphPad Software) for statistical analyses. Values represent the mean ± standard deviation (S.D.). Statistical significance was determined based on *p*-values obtained from an unpaired two-tailed Student’s *t*-test or two-way ANOVA.

## Electronic supplementary material

Supplementary Figure S1

Supplementary Figure S2

Supplementary Figure S3

Supplementary Figure S4

Supplementary materials

Supplementary figure legends
