# Supplementary figures and images for: The cisplatin-induced lncRNA PANDAR dictates the chemoresistance of ovarian cancer via regulating SFRS2-mediated p53 phosphorylation
Source: Cell Death Dis. 2018 Oct 30;9(11):1103. doi: 10.1038/s41419-018-1148-y (PMC6207559; doi:10.1038/s41419-018-1148-y)

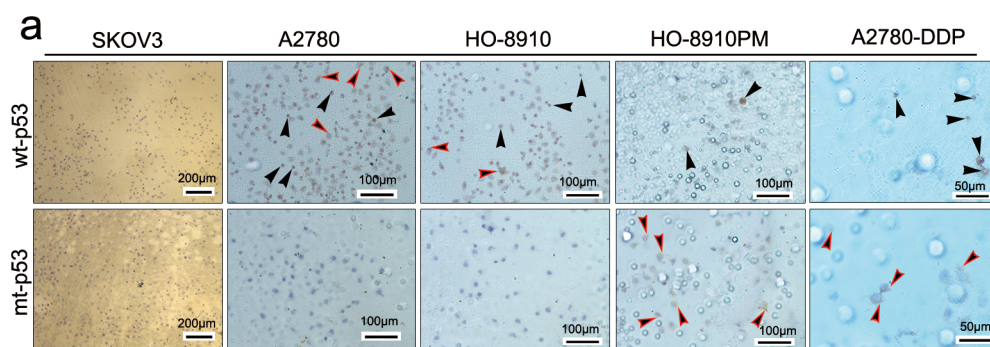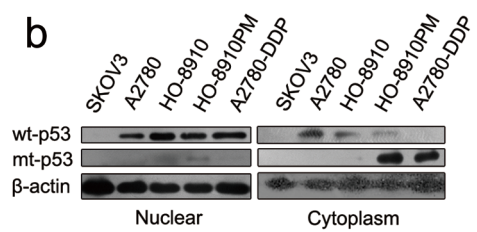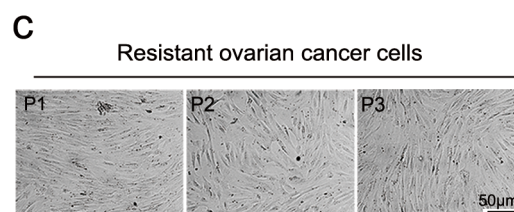

Supplement: Supplementary file 1 — Supplementary Figure S1 [file 41419_2018_1148_MOESM1_ESM.pdf]

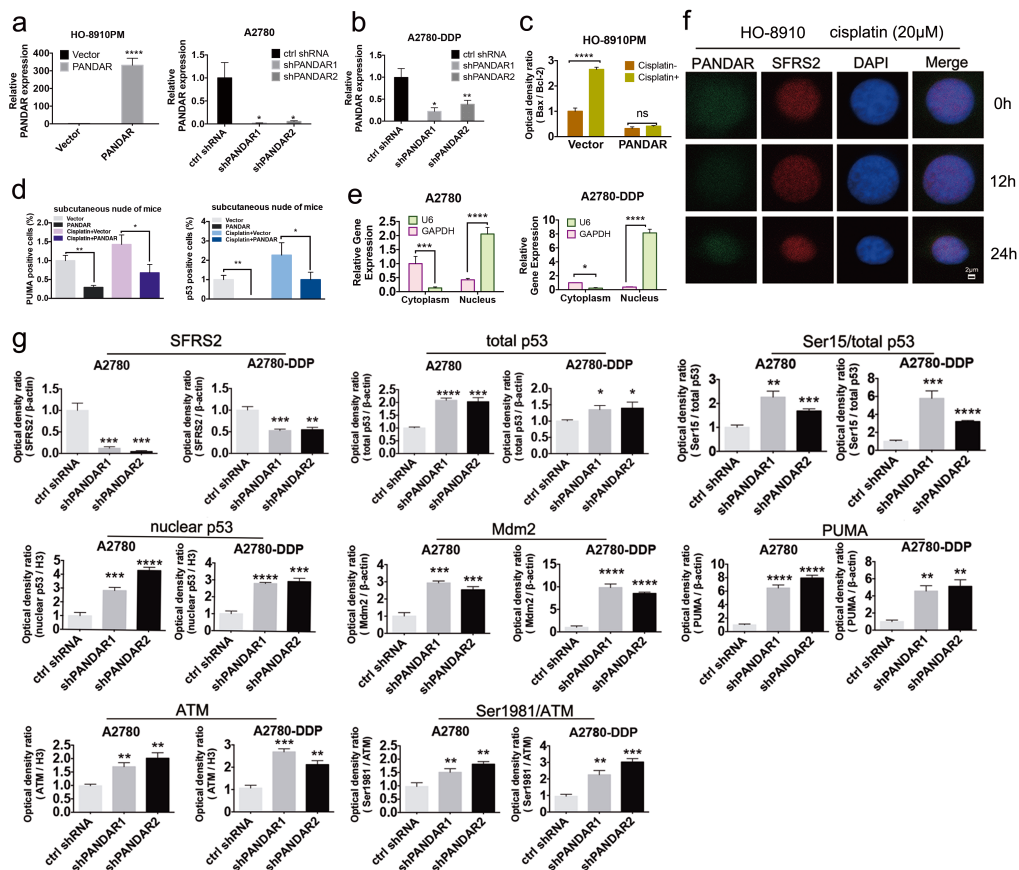

Supplement: Supplementary file 2 — Supplementary Figure S2 [file 41419_2018_1148_MOESM2_ESM.pdf]

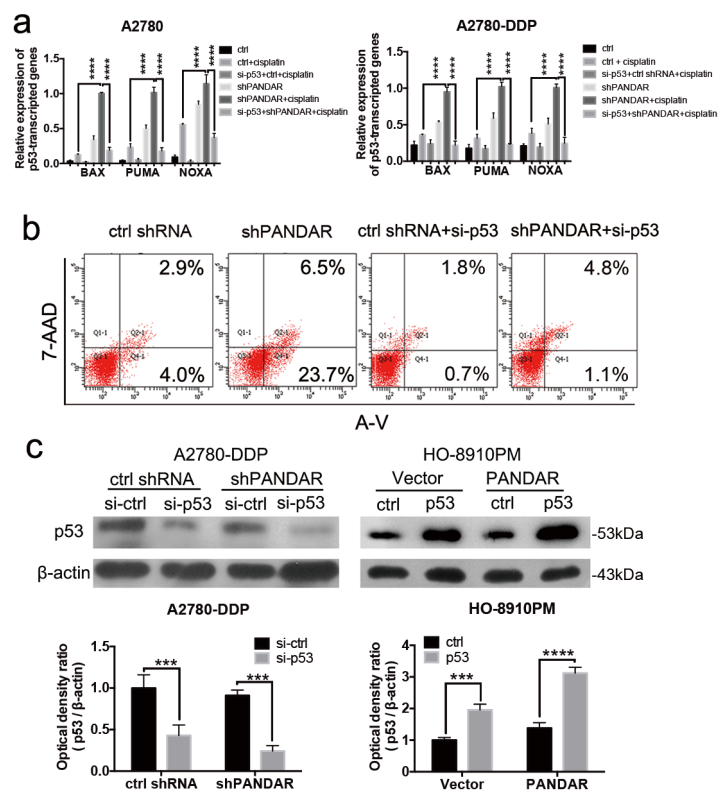

Supplement: Supplementary file 3 — Supplementary Figure S3 [file 41419_2018_1148_MOESM3_ESM.pdf]

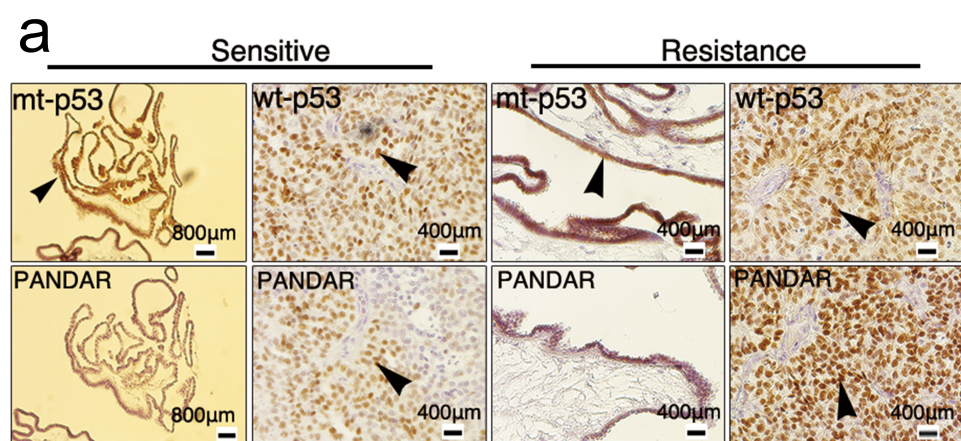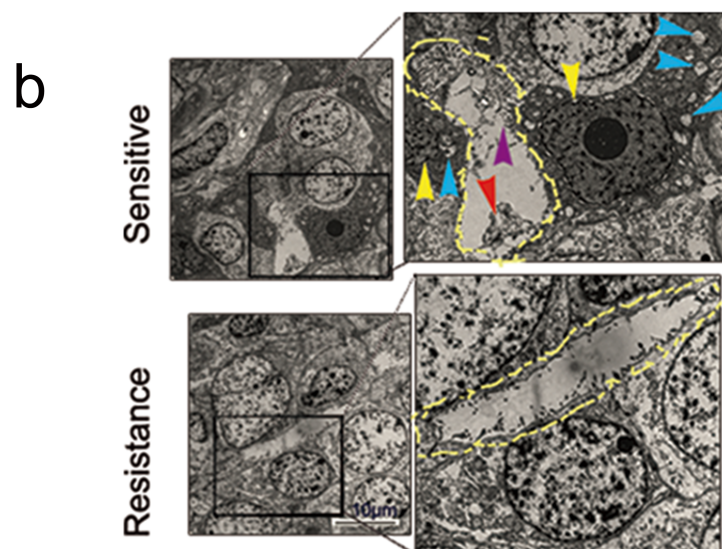

Supplement: Supplementary file 4 — Supplementary Figure S4 [file 41419_2018_1148_MOESM4_ESM.pdf]
