# Supplementary material for: The cisplatin-induced lncRNA PANDAR dictates the chemoresistance of ovarian cancer via regulating SFRS2-mediated p53 phosphorylation
Source: Cell Death Dis. 2018 Oct 30;9(11):1103. doi: 10.1038/s41419-018-1148-y (PMC6207559; doi:10.1038/s41419-018-1148-y)

**Supplementary** **Materials**

**Supplementary Tables**

| Supplementary Table 1. Reagents and antibodies | | | |
| --- | --- | --- | --- |
| **Reagents / antibodies** | Code | Origin | physical characters |
| Paditaxel | SP8020 | Solarbio(Beijing,China) | powder( dissolved in ddH2O) |
| Doxorubicin(Dox) | D8740 |  | powder(dissolved in ddH2O) |
| Dimethyl sulfoxide(DMSO) | D8370 | Siga-Aldrich (St Louis, MO, USA) | liquid |
| anti-AKT2 | L79B2 | Cell Signaling Technology | liquid |
| anti-PUMA | ab33906 | Abcam | liquid |
| anti-Bcl-2 | D55G8 | Cell Signaling Technology | liquid |
| anti-Bax | D2E11 | Cell Signaling Technology | liquid |
| anti- p53 | 7F5 | Cell Signaling Technology | liquid |
| Phospho-p53 (Ser15) Antibody | 9284 | Cell Signaling Technology | liquid |
| Phospho-p53 (Ser20) Antibody | 9287 | Cell Signaling Technology | liquid |
| ATM Antibody | D2E2 | Cell Signaling Technology | liquid |
| Phospho-ATM (Ser1981) | D25E5 | Cell Signaling Technology | liquid |
| anti-β-actin | TA-09 | ZSBH (Beijing,China) | liquid |
| Histone H3 | 96C10 | Cell Signaling Technology | liquid |
| Dimethyl sulfoxide(DMSO) | D8370 | Siga-Aldrich (St Louis, MO, USA) | liquid |
| SFRS2 (SC35) | ab204916 | Abcam | liquid |
|  |  |  |  |

| Supplementary Table 2. Primer sequences and gene related sequences | | | | | | |
| --- | --- | --- | --- | --- | --- | --- |
| RT-PCR primers (human) | Primer sequence | | **Annealing temperature** | Name/Gene ID(human) | **Location(human)** | Aliases |
|  | **Forward** | **Reverse** |  |  |  |  |
| GAPDH | 5’ -AGCCACATCGCTCAGACAC-3’ | 5’ -GCCCAATACGACCAA-3’ | 60℃ | ID: 2597 | NC_000012.12 | G3PD, GAPD, HEL-S-162eP |
| U6 | 5’-GCTTGCTTCAGCAGCACATA-3’ | 5’ -AAAAACATGGAACTCTTCACG-3’ | 60℃ | ID: 1497008 | NC_015438.2 | None |
| PANDAR | 5’-TGCACACATTTAACCCGAAG -3’ | 5’-CCCCAAAGCTACATCTATGACA-3’ | 60℃ | ID: 101154753 | NC_000006.12 | PANDA |
| PUMA | 5’-GTGTGGAGGAGGAGTGG-3’ | 5’-TCGGTGTCGATGTTGCTCTT-3’ | 58.7℃ | ID: 27113 | NC_000019.10 | JFY-1, JFY1, BBC |
| BAX | 5′- GCATCCACCAAGAAGCTGAG-3′ | 5′-CAAAGTAGAAGAGGGCAACC -3′ | 58.7℃ | ID: 581 | NC_000019.10 | BCL2L4 |
| NOXA | 5’-TCCTGAGCAGAAGAGTTTGG-3’ | 5’-GGAGATGCCTGGGAAGAAGG-3’ | 60℃ | ID: 5366 | NC_000018.10 | PMAIP1, APR |
| Gene Knock-down | Sequences |  |  |  |  |  |
| shPANDAR 1 | 5‘-AATGTGTGCACGTAACAGA-3’ |  |  |  |  |  |
| shPANDAR 2 | 5‘-GGGCATGTTTTCACAGAGG-3’ |  |  |  |  |  |
| shSFRS2 | 5’-GCGUCUUCGAGAAGUACGGTT-3’ |  |  |  |  |  |
| Gene probe | Forward | **Reverse** |  |  |  |  |
| PANDAR | CTGCCCAGAAGCAAACAGGACTC | TTTGGGAGACCGAGGCAGACAGA |  |  |  |  |
| Gene Silencing | Sense | Antisense |  |  |  |  |
| si-p53 | CACCUCACUGCAUGGACGAUCUGUU | AACAGAUCGUCCAUGCAGUGAGGUG |  |  |  |  |

***Gene Probe***

LncRNA PANDAR-F: CTGCCCAGAAGCAAACAGGACTC

LncRNA PANDAR-R: TTTGGGAGACCGAGGCAGACAGA

LncRNA PANDAR Sequence:

ACGAATTCTTTCAGGAATGCCGCAGATGTACATGCTCCCGCAGATCTATATTTTCCAATGTTGTTAACATCAGCCAGCTGGCAATCTACAACCTGTCTTGTACAATGTTTGAAGAGAGGCATCCTCCAGACACGGTCCCCTGTTTCAATGCTGGCCTCGAAGAGCTTGTTCCAGAGCCAGGATGAATTGGTAAAGACCCCAGTGGCACCTGACCCCAAAGCTACATCTATGACACCTGTTAAGGTGGTGGCATTGAGGATGACCTTCGGGTTAAATGTGTGCACGTAACAGAGCGCATCAGCCAGTATGAGCCTCCCCTCAGCATCAGTGTTACCAACCTGGATGGTCTTCCTGTTCCTGGCTCTAACAACATCCCCCAGCTTGTTGGCCTTGCCGCTGGGCATGTTTTCACAGAGGGGCCAGACCTATAATATTAATGGGCAAACTGAGATTTGCAGCAGACACAATGGCTGAGCATATAGTTGTAGCTCCTCCCATGTCGGCCCTCATGAGGTCCATATTTGCAGAAGCCTTGATGGAGATACCACCACTGTCAAAGGTAATTCCTTTCCCAACAAACAAGGGGTGGTTTGTCTGCATTGGGGCTGCCTATGTAGTGAATTTCCAAGAAGACTGAGGGCTCGTCAGATCCTTTGGCCACACTGAGGAATGATCCCATTGCCTGTTCCTCAATCCAAGACCTGGGTCTGATATGAAACTCGGTTTACTACTAGCGCTTTTGAGATTCTTCTCAATAATTTCGGCAAATCTGGTTGGCATCATCTCGCTGGCTGGCGTCTCCATCATGCCAAGTTCTGCCCAGAAGCAAACAGGACTCCTTTCTGCCAGGCCTCCTGATCCCCAGTTCCATAGAGCTTCACCGACATAGCCATCTTCTTTTTTTGCTTTAGGTCATCGTATTCATAGAGACCAAGCACCGCGCCCTCCTCAGCAGCCTGAGCATCTCTACAGGGATCCACCTCCACGGAAGAGAGCTCCAGGTCTTGAATCTGCCTGCATCCTGCTGCAACAGCAGCTCTGATGTTTTCTTTGCCTTCCTGCCAGTTTTCCTGTTCGTCGATTCTGGCTGCCTTTTTGCCGAGGCCAACTAGCACCACGCTGGGGAAGTCCTGATGCAGACCATAAAAGTTTCGAGTCTTGCCTGCCTTCAGAGGTGGTCCAGATATGTTCAAAGTCTCTCTCAGCTTTCCAGCTATCAATTTATCAAGATTCTCTCCTGCACTTGTGAACTGTGGCACATCATCTTCTTTTTCTTTGGAATAGATTCCTAAAACAAGGCCCTTCGTCATGTCTGCAGCGGAGAGACCCCGGCTCCCGAAACATCTCACGGCCAGACGTCAGACGATGACTCGCCCCACCCAATGTTTGTATTTTAGTAGAGAGAGGGTTTCTCTATGTTGGTCAGGCTGGTCTCAAACCTCGACCTCAGTTGATCTGTCTGCCTCGGTCTCCCAAAGTGCTGAGATTACAGGCGTGAGCCACTGC

**Predicted PANDAR-binding proteins in RBPDB Database**

([*http://rbpdb.ccbr.utoronto.ca/cgi-bin/sequence_scan.pl*](http://rbpdb.ccbr.utoronto.ca/cgi-bin/sequence_scan.pl))

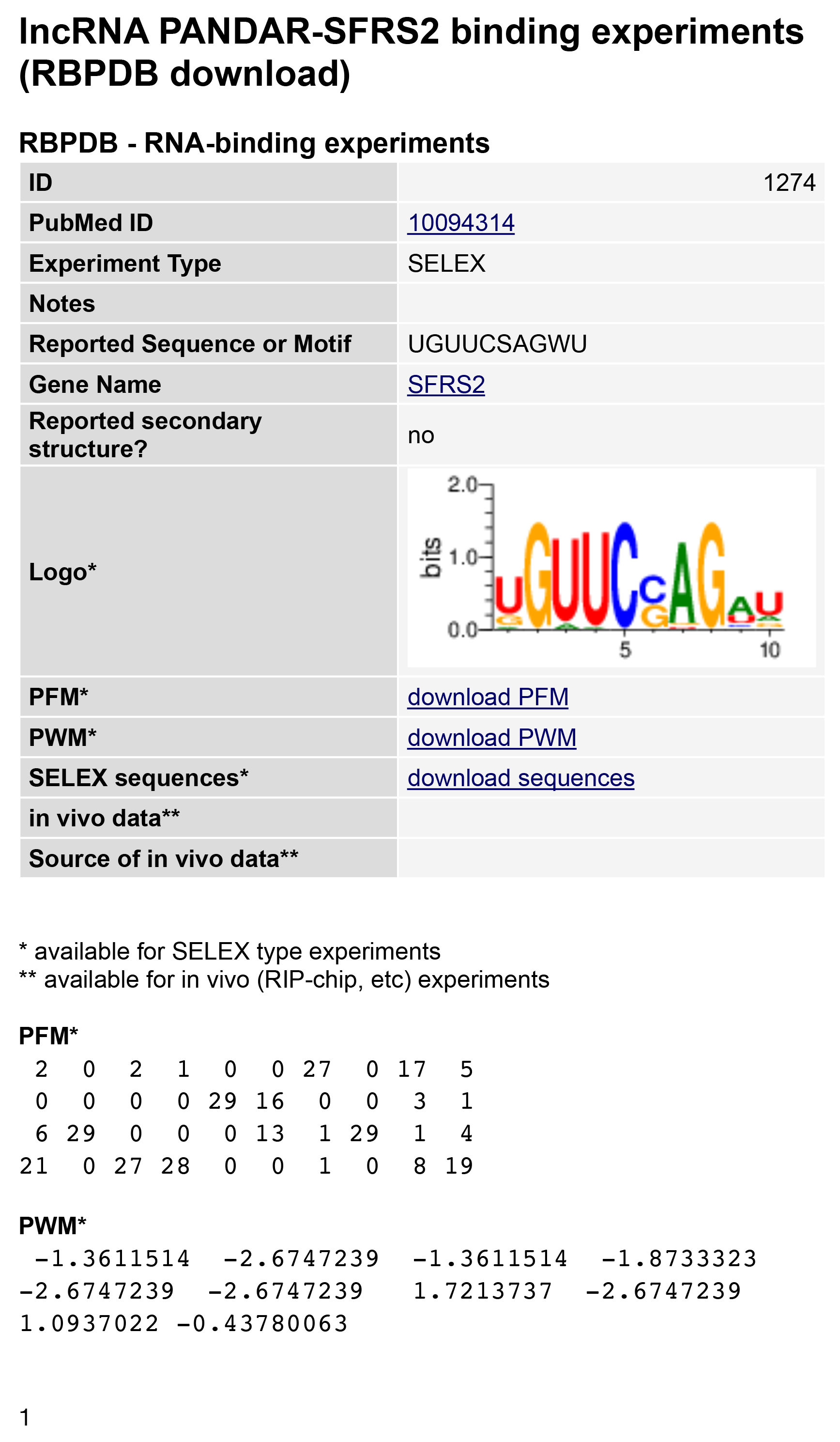

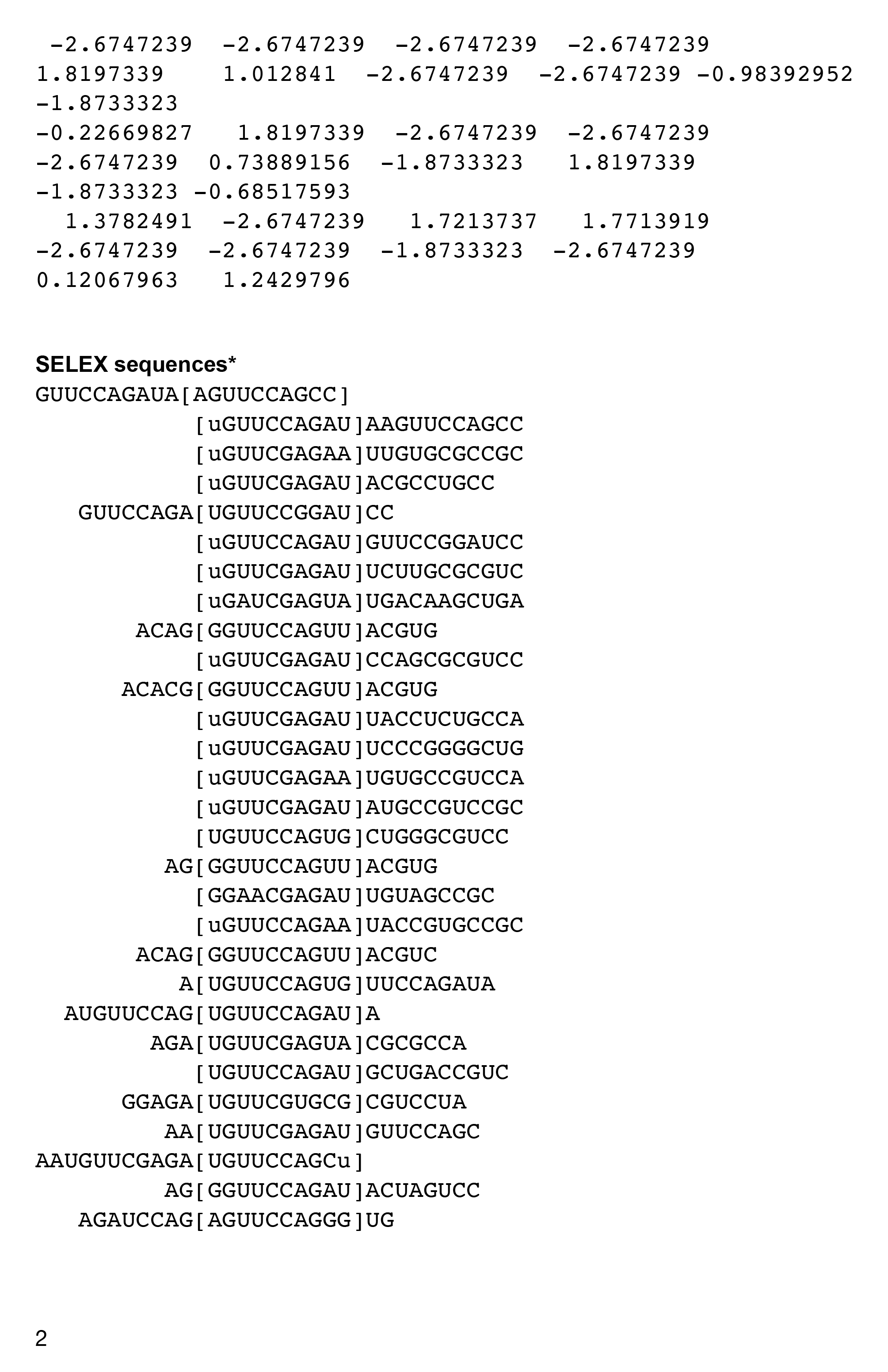

Supplement: Supplementary file 5 — Supplementary materials [file 41419_2018_1148_MOESM5_ESM.docx]
