# Supplementary material for: The cisplatin-induced lncRNA PANDAR dictates the chemoresistance of ovarian cancer via regulating SFRS2-mediated p53 phosphorylation
Source: Cell Death Dis. 2018 Oct 30;9(11):1103. doi: 10.1038/s41419-018-1148-y (PMC6207559; doi:10.1038/s41419-018-1148-y)
Supplement: Supplementary file 6 — Supplementary figure legends [file 41419_2018_1148_MOESM6_ESM.docx]

**Supplementary** **Figure** **legends**

**Figure S1.** **Expression of wild-type and mutant p53 in ovarian cancer cell lines**

1. Immunocytochemistry of wild typ53 and mutant p53 expression performed with specific antibodies in ovarian cancer cisplatin-sensitive (SKOV3, HO-8910, HO-8910PM, A2780) and -resistant (A2780-DDP) cell lines. Black arrow heads indicate nucleus positive expression and red border arrow heads indicate cytoplasmic positive expression. Scale bar: 50μM,100μM, 200μM.
2. Protein expression of wild type p53 and mutant p53 in the nuclear and cytoplasm fractions from ovarian cancer cell lines, separately.
3. Primary cells of recurrent ovarian cancer patients. Passage 1 (P1), 2 (P2), 3 (P3) primary cells isolated from patients with recurrent ovarian cancer. Scale bar: 50 μm.

**Figure S2. PANDAR and its related proteins expression in ovarian cancer cells.**

(a, b) PANDAR overexpression in HO-8910PM cells (a) and knockdown in A2780 (a) and A2780-DDP cells (b) after transfected with lentiviral plasmids containing PANDAR or PANDAR-shRNA sequence.

(c) According quantification of BAX/Bcl-2 ratio (Fig. 2h) via western blotting assay in HO-8910PM-PANDAR overexpressing cells after treated with 20μM dose of cisplatin for 12 h. Non-treated cells serve as controls.

1. According positive cell counting of p53 (right) (Fig. 3f) and PUMA (left) (Fig. 3g) in immunofluorescent staining of mice tumor formed with HO-8910PM-PANDAR or -Vector cells with or without cisplatin treatment. Non-treated mice with HO-8910pm-Vector cells serve as controls.
2. QRT-PCR of U6 and GAPDH gene expression in cellular nuclear and cytoplasm isolated from A2780 and A2780-DDP cells, separately. U6 serves as nuclear control. GAPDH serves as cytoplasm control.
3. Fluorescence in situ hybridization (FISH) assay of PANDAR RNA (green) in HO-8910 cells distributes in discrete foci through the nucleus and cytoplasm at the beginning of cisplatin treatment (0h) with dosage of 20μM. And the foci increased and focused in the nucleus after cisplatin treatment for 12h and 24h. Co-localization of SFRS2 protein (red) in HO8910 cells via immunocytochemistry performed with SFRS2 antibody and showed an increasing discreted foci through the nucleus after cisplatin treatment (12h, 24h), and is not detected in cytoplasm. Scale bar: 2 μm.
4. According quantification of proteins expression in Fig 5f. Ctrl shRNA cells serve as controls.

Vector and control groups are normalized to 1. Data presents the mean ± S.D. n = 3 independent experiments. * p<0.05, **p<0.01, ***p<0.001, ****p<0.0001, ns, non-significant, unpaired two-tailed Student’s *t-*test.

**Figure S3. PANDAR downregulated p53-related genes expression and p53-mediated apoptosis**

1. Expression levels of *BAX, PUMA,* and *NOXA* in A2780-(left) and A2780-DDP-(right) PANDAR-knockdown cells (shPANDAR) with or without p53 silenced after treated with cisplatin and measured by qRT-PCR. Non-treated cells serve as controls. (mean ± S.D. n = 3) *p<0.05, ***p<0.001, ****p<0.0001, determined by Student’s *t*-test.
2. Flow cytometry assay of apoptosis measurement in A2780-DDP-PANDAR knockdown (shPANDAR) cells with or without p53 silenced after incubated with 20 μmol/L dose of cisplatin for 24 h. Early apoptotic population in the lower right gate is characterized with Annexin V (+) and 7-AAD (-), and late apoptotic population in the upper right gate is characterized with Annexin V (+) and 7-AAD (+), showing that shPANDAR upregulated apoptosis was p53-dependent.
3. SiRNA of p53 in A2780-DDP PANDAR-knockdown (shPANDAR) or control cells (ctrl shRNA) and p53 overexpression in HO-8910PM-PANDAR or -Vector cells were assessed by western blotting assay (up) and accordingly quantification (down).

Control or Vector cells are normalized to 1. Data presents the mean ± S.D. n = 3 independent experiments. ***p<0.001, ****p<0.0001 determined by Student’s *t*-test.

**Figure S4.** **PANDAR expression and cell apoptosis pattern in clinical specimens from relapsed ovarian cancer patients.**

1. LNA ISH analysis of lncRNA PANDAR with LNA probes and IHC assay of wild type p53 and mutant p53 with specific antibodies in matched ovarian cancer tissues before platinum-based therapy (sensitive) and after disease progression during platinum-based treatment (resistance) (Accordingly quantification see Fig. 6a). Representative LNA ISH and IHC images are shown. Black arrow head indicates the positive expression of lncRNA or protein. Scale bar: 400 μm, 800 μm.
2. TEM imaging of the injured cancer cells characterized with nuclear membrane shrinkage (yellow arrow heads), cytoplasm shrinkage and vacuolization (blue arrow heads), glandular cavity (yellow dashed circle) discontinuity (purple arrow heads), while tissues from chemoresistant patients exhibited with no shrinkage in nucleus or cytoplasm, and the glandular cavity (yellow dashed circle) in resistant tissue is completely continuous. Scale bar: 10 µm. Mean ± S.D. n=7 independent fields per group.
